# Modeling SNP array ascertainment with Approximate Bayesian Computation for demographic inference

**DOI:** 10.1038/s41598-018-28539-y

**Published:** 2018-07-05

**Authors:** Consuelo D. Quinto-Cortés, August E. Woerner, Joseph C. Watkins, Michael F. Hammer

**Affiliations:** 1 National Laboratory of Genomics for Biodiversity (LANGEBIO), CINVESTAV, Irapuato, 36821 Mexico Mexico; 2 Center for Human Identification, University of North Texas Health Science Center, Texas, 76107 USA; 3 Department of Mathematics, University of Arizona, Tucson, Arizona 85721 USA; 4 ARL Division of Biotechnology, University of Arizona, Tucson, Arizona 85721 USA

## Abstract

Single nucleotide polymorphisms (SNPs) in commercial arrays have often been discovered in a small number of samples from selected populations. This ascertainment skews patterns of nucleotide diversity and affects population genetic inferences. We propose a demographic inference pipeline that explicitly models the SNP discovery protocol in an Approximate Bayesian Computation (ABC) framework. We simulated genomic regions according to a demographic model incorporating parameters for the divergence of three well-characterized HapMap populations and recreated the SNP distribution of a commercial array by varying the number of haploid samples and the allele frequency cut-off in the given regions. We then calculated summary statistics obtained from both the ascertained and genomic data and inferred ascertainment and demographic parameters. We implemented our pipeline to study the admixture process that gave rise to the present-day Mexican population. Our estimate of the time of admixture is closer to the historical dates than those in previous works which did not consider ascertainment bias. Although the use of whole genome sequences for demographic inference is becoming the norm, there are still underrepresented areas of the world from where only SNP array data are available. Our inference framework is applicable to those cases and will help with the demographic inference.

## Introduction

One of the main goals of the HapMap project was to describe common patterns of variation in the human genome, initially from individuals with African, European and Asian descent^1^. This effort led to the characterization and selection of sequence variants or single nucleotide polymorphisms (SNPs), that were later included in commercial genotyping arrays. However, since another goal of this project was to establish associations between these variants and common diseases, most of the SNPs were confined to those of intermediate frequency in genic regions, therefore creating a representation bias. Operating under the “common disease, common variant” model, SNPs with these characteristics were selected to increase the power of association tests^2^. Once array data became available from several populations, they were used for a different purpose: inference of population genetic or demographic parameters. Unfortunately, the approach used to identify variants for these SNP arrays may adversely affect the quality of such inferences.

When SNPs are found in a small sample of individuals from selected populations and then genotyped in other populations, sites with intermediate frequencies in the discovery panel will be reliably scored in other populations, while variants that are rare or absent in the discovery panel will not be accurately accounted for when they are common (or rare) in the population of interest. As a consequence, the observed patterns of nucleotide diversity will be skewed^3,4^. This phenomenon is known as ascertainment bias, which results in the deviation of population genetic statistics and in the loss of important genetic information specific to the history of a population^5^. This deviation is particularly relevant as several available human datasets derived from genotyping arrays have been regularly used for demographic inference^6^. Inference for population genetic studies usually rely on the site frequency spectrum and other summary statistics to study demography. Thus an excess of common sites leads, for example, to the underestimation of the overall heterozygosity and FST, and the overestimation of Tajima’s D^4,5,7–9^.

While complete sequencing of all samples in a study eliminates the effect of ascertainment bias, this option is not always available given the much higher cost relative to SNP array genotyping and there are still populations of special interest from which only SNP data are available. In addition, ancient DNA sequences have been enriched for a panel of known SNPs which, in turn, introduces ascertainment bias in the analyses of this type of samples^10,11^.

To address these ascertainment bias issues, several methods have been proposed to correct the site frequency spectrum generated by the SNP array data^3,4,8,9,12^, or to directly account for the underlying bias in the arrays^13^. Some of these methods depend on knowing exactly the methodology used to determine SNPs, which is rarely known for available arrays. For example, in the original phase of the HapMap project, the criteria for selecting variable sites changed during the course of the SNP discovery process^4^, making methods for correction even more challenging. Indeed, investigators have acknowledged that these methods are difficult to generalize across studies^9^.

In this study, we develop an inference pipeline that explicitly models the SNP discovery protocol by varying the haploid sample size and the site frequency cut-off. We recreate the SNP distribution of two different Affymetrix arrays and use a bootstrap strategy to jointly estimate the ascertainment and demographic parameters in an Approximate Bayesian Computation framework^13,14^. We apply this method to estimate parameters of the Out-of-Africa model and of the admixture model of the Mexican population. This methodology has the capacity to address diverse demographic questions when only SNP array data are available and is applicable to different array platforms.

## Methods

### Sample collection

In this study we investigated the underlying ascertainment of the Affymetrix Axiom Genome-Wide Human Array (Axiom array) data and the admixture dynamics of the modern Mexican population (Supplementary Table S1).

We gathered high coverage whole genomes of unrelated Yoruba (YRI, *n* = 9), Utah residents with European ancestry (CEU, *n* = 9), and Han Chinese (CHB, *n* = 4) sequenced by Complete Genomics (CGI genomes)^15^. This was matched with Axiom SNP array data from the same YRI, CEU and CHB individuals. To infer the time of contact of Europeans with the indigenous populations in Mexico, we collected data from individuals with Mexican ancestry (MXL, *n* = 104) and from Iberian Populations in Spain (IBS, *n* = 162), all part of the 1000 Genomes Project^16^. We also used previously published genotypes from the Nahuas of Puebla (NXP, *n* = 22)^17^. All three populations were genotyped on the Affymetrix Genome-Wide Human SNP Array 6.0 (Affy 6.0).

### Modeling ascertainment bias

**Figure 1:**
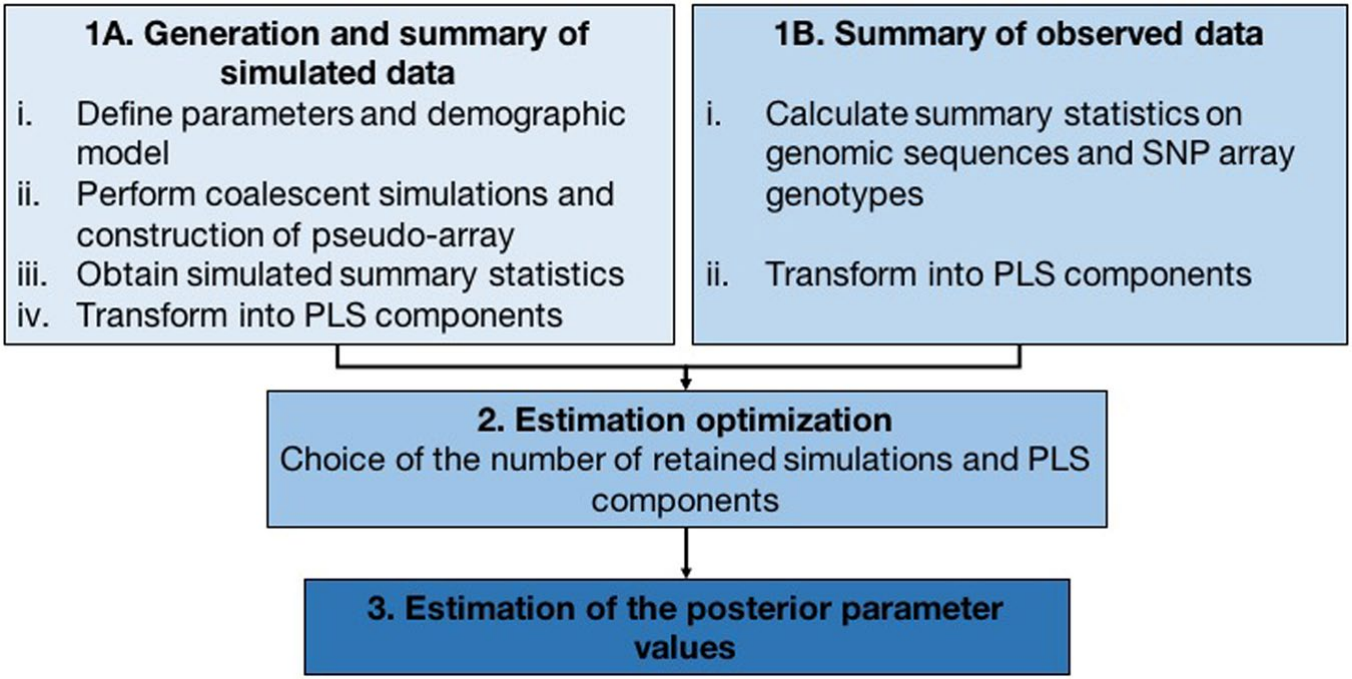
Flow chart of our inference method.

We describe the pipeline in the following sections and a diagram is depicted in Fig. 1. The main purpose of this pipeline is to model the ascertained data present in the array (in this case, SNPs) and unascertained data, and to use summary statistics on both datasets to estimate parameter values with ABC.

Using the Neutral Regions Explorer^18^, we determined a set of 1386 10 kb genomic neutral regions in order to reduce the interference of natural selection on demographic inference. This tool allows the rapid application of several criteria to find neutral regions in the human genome. Our filters were the exclusion of genes, segmental duplications and other repeats, copy number variants, and an interval of 0.1 cM between non-overlapping regions. The final set of regions is herein referred as ‘10 kb loci’.

**Figure 2:**
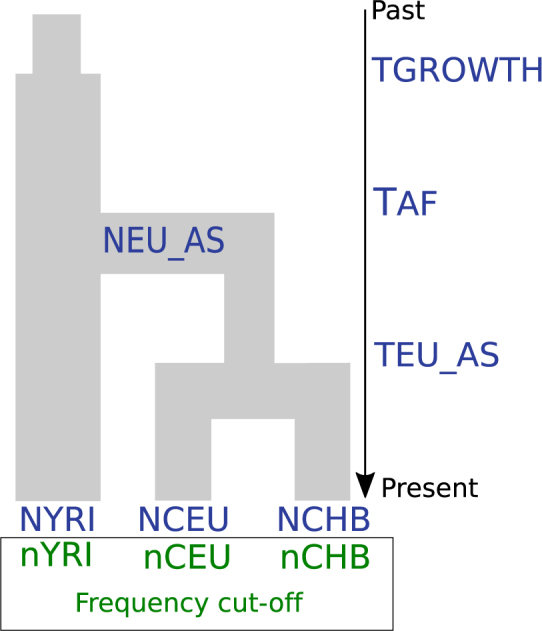
Out-of-Africa model with HapMap populations. This model includes the effective population sizes of sub-Saharan Yoruba (NYRI), European (NCEU) and Chinese (NCHB) populations; as well as the ancestral population size of Europeans and Asians after the Out-of-Africa (NEU_AS). The time of divergence between Europeans and Asians (TEU_AS), the time of split between Africans and non-Africans (TAF) and the time of population expansion in Africans (TGROWTH) are also part of this model. The parameters of the discovery set (haploid sample size in each population and the frequency cut-off) are marked in green, inside the box. See Fig. 4 for the posterior estimates of the parameters.

We assumed that the YRI, CEU and CHB populations, as part of Phase I of the HapMap project, were the first populations genotyped and that the variable sites discovered in these populations are the basis for most of the currently available SNP arrays^1^. We then simulated a simple demographic model of the divergence of the HapMap populations (Fig. 2) with a small number of parameters. We excluded demographic events like exponential population growth and migration in these populations as opposed to other studies^19,20^. We note that demographic events like the aforementioned influence the genetic diversity of populations and therefore the detection of variant sites to be potentially included in a SNP array. Nonetheless, we verified that this simplified model produced summary statistics with similar values to the observed summary statistics (See Discussion).

A set of values was drawn for the prior distribution of the parameters, both demographic and ascertainment, and one unique simulation was performed with that set of values. In this manner, we can jointly infer all the parameters of the model. The parameters of the model and their priors are shown in Table 1. We used a customized version of the simulator MaCS (Markovian Coalescent Simulator)^21^ for Python, which allows faster processing of the coalescent simulations. We performed one million simulations of the whole set of 10 kb loci, according to the demographic scenario in Fig. 2. In addition to the demographic parameters, we included four SNP ascertainment parameters in the simulations: the haploid sample size from each of the three HapMap populations to select variable sites (what we call discovery set) and a minor allele frequency cut-off to determine a SNP in this discovery set (Step 1A of pipeline).

**Table 1:** Prior and posterior distributions of the Out-of-Africa model.

Parameter	Prior distribution		Distribution	Mode	Posterior estimation	
					HPDI 95	
	Minimum	Maximum			Lower	Upper
nYRI	2	20	Discrete uniform	3.82	2	9.59
nCEU	2	20	Discrete uniform	17.82	4.58	20
nCHB	2	20	Discrete uniform	17.09	6.5	20
Frequency cut-off	0.05	0.10	Uniform	0.094	0.07	0.10
log10 (NYRI)	3.7	5.0	Uniform	4.63	4.36	4.90
log10 (NCEU)	3.0	5.0	Uniform	4.62	4.28	4.95
log10 (NCHB)	3.0	5.0	Uniform	3.67	3.52	3.79
NEU_AS	1500	5000	Uniform	4469.7	2216.22	5000
TEU_AS	200 (5,000)	1599 (39,975)	Discrete uniform	1401.16 (35,029)	1160.94 (29,024)	1599 (35,975)
TAF	1600 (40,000)	4100 (102,500)	Discrete uniform	2786.87 (69,672)	2117.68 (52,942)	3277.96 (81,949)
log10 (NANC)	0.0	−1.0	(10** Uniform (−1.0,0.0)) *NYRI)	—	—	—
TGROWTH	1	4100	Discrete uniform	—	—	—

Parameter labels correspond to those given in Fig. 1. The effective population sizes are set to a log10 scale and divergence times are given in generations units (25 years) and in years (values in parenthesis). For posterior distributions, see Supplementary Fig. S2. Log(NANC) corresponds the ancestral population size in Africans while TGROWTH is the time of population growth.

We established the following strategy to model the SNP ascertainment process. For each locus in the 10 kb loci from the aforementioned coalescent simulations, we created a “pseudo-array” with the same number of sites as the Axiom array and with a similar physical distance distribution between the SNPs. To accomplish this, we randomly selected from the prior distribution a number of haploid samples from each of the HapMap populations and simulated segregating sites. In this discovery set, sites with frequency greater than the cut-off were selected and, of these available sites, sites with the closest physical position to the corresponding Axiom SNPs were kept for subsequent analysis. A simulation was repeated until sufficiently many segregating sites were obtained (Step 1 A of pipeline). One simulation included: the simulation of the whole genome data (10 kb loci) in the sample that includes 9 YRI, 9 CEU and 4 CHB diploid individuals, of the discovery panel and of the ascertained data (ascertained SNPs in each of the 10 kb loci in the discovery panel).

The summary statistics calculated in the simulated (Step 1 A) and the observed data (Step 1B) were later transformed into Partial Least Squares components in order to obtain linear combinations of the summary statistics. We calculated the mean and standard deviation of site frequency spectrum and haplotype based summary statistics across the 10 kb loci extracted for the CGI genomes and for the Axiom SNPs that are present in those loci. The summary statistics used were the number of segregating sites, singletons, doubletons, Tajima’s D, number of distinct haplotypes and number of the most frequent haplotype per population, FST and number of shared and private haplotypes between pairs of populations.

We inferred the ascertainment and demographic parameters for the Out-of-Africa model with the software ABCtoolbox^22^ and to optimize the parameter estimation, we varied the number of PLS components and the number of retained simulations (Step 2). For each choice, we re-sampled the one million simulations with replacement and used ABCtoolbox to estimate the parameters. This step was repeated 1000 times for each choice. We then computed the covariance matrix of the posterior estimates (to maintain the structure of the joint estimation of the parameters) and simulated 1000 random values using its covariance structure.

We then performed simulations using these 1000 parameter values, standardized the observed and simulated summary statistics and calculated the Euclidean distance between them. Lastly, we selected the number of PLS components and number of retained simulations that minimized this distance and confirmed that its parameter values generated summary statistics like the observed ones. We then retained the posterior mode and 95% High Posterior Density Intervals (HPDI) for each parameter (Step 3), as estimated by ABCtoolbox. For more details on the different steps of the methodology, refer to the supplemental materials.

The list of the 10 kb loci and all the scripts used in this work can be found in the following link: https://bitbucket.org/cdquinto/ascertainment-bias-scripts/.

### Mexican admixture example

We examined the admixture process giving rise to the present-day Mexican Mestizo population with the proposed demographic pipeline. We used the data described above from the HapMap, IBS, MXL and NXP populations.

**Figure 3:**
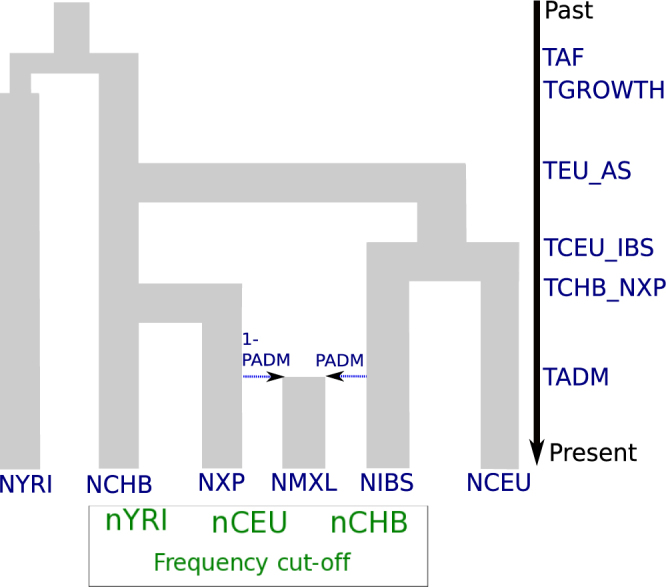
Admixture model involving the Mexican population. This model includes the parameters from the Out-of-Africa model (Fig. 1), the effective population sizes of the Nahuas (NXP), Mexican (NMEX) and Iberian (IBS) populations. The times of divergence between Asian and Native Americans (TCHB_NXP) and between Northern and Western Europeans and Iberians (TCEU_IBS) populations are also included. The admixture parameters are the time (TADM) and the European contribution (PADM) to the indigenous population. The parameters of the discovery set are written in green, inside the box. See Fig. 5 for the posterior estimates of the parameters.

We established the model shown in Fig. 3 as a simplified depiction of this admixture process. We used the NXP and IBS populations as the principal contributors and assumed that these populations diverged from one of the HapMap populations. This model includes the parameters of the Out-of-Africa model as well as the time and proportion of admixture (Table 2). We performed one million simulations of this model and followed the pipeline described above to estimate the parameter values, with some additional steps. The discovery set parameters were used to determine the ascertained sites for the Affy 6.0 array in the HapMap samples and summary statistics of the IBS, MXL and NXP chromosomes were calculated at these specific SNPs for each region in the 10 kb loci. As a consequence, the final set of simulated summary statistics included summary statistics from the 10 kb loci set and ascertained sites for the HapMap populations, and from only the ascertained sites for the IBS, MXL and NXP populations.

**Table 2:** Prior and posterior distributions of the Mexican admixture model.

Parameter	Prior distribution		Distribution	Mode	Posterior estimation	
					HPDI 95	
	Minimum	Maximum			Lower	Upper
nYRI	2	20	Discrete uniform	6.91	2	17.39
nCEU	2	20	Discrete uniform	16	3.56	19.91
nCHB	2	20	Discrete uniform	7.45	2	17.58
Frequency cut-off	0.05	0.10	Uniform	0.061	0.051	0.085
log10 (NYRI)	3.7	5.0	Uniform	4.44	4.1	4.99
log10 (NCEU)	3.0	5.0	Uniform	4.09	3.7	4.64
log10 (NCHB)	3.0	5.0	Uniform	3.55	3.27	3.68
NEU_AS	1500	5000	Uniform	4151.52	2190.93	4982.32
TEU_AS	500 (12,500)	1599 (39,975)	Discrete uniform	1410.28 (35,257)	1055.98 (26,400)	1599 (39,975)
TAF	1600 (40,000)	4100 (102,500)	Discrete uniform	1953.54 (48,839)	1600 (40,000)	2493.52 (62,338)
TGROWTH	1	4100	Discrete uniform	—	—	—
log10 (NNXP)	3.0	5.0	Uniform	4.13	3.38	4.91
log10 (NIBS)	3.0	5.0	Uniform	4.72	3.4	5
log10 (NMXL)	3.0	5.0	Uniform	4.21	3.31	5
TCEU_IBS	400 (10,000)	TEU_AS	Discrete uniform	976 (24,400)	400 (10,000)	1297.33 (32,433)
TCHB_NXP	400 (10,000)	TEU_AS	Discrete uniform	688.73 (17,218)	418.05 (10,451)	1491.44 (37,286)
TADM	16 (400)	24 (600)	Discrete random	21.5 (538)	16.53 (413)	24 (600)
PADM	0.0	1.0	Uniform	0.53	0.43	0.65

Parameter labels correspond to those given in Fig. 2. The effective population sizes are in a log10 scale and divergence times are given in generations units (25 years) and in years (values in parenthesis). For posterior distributions, see Supplementary Fig. S5. Log(NANC) corresponds the ancestral population size in Africans while TGROWTH is the time of population growth.

### Validation and comparison of the pipeline

To validate this method we used simulated summary statistics with their associated known parameter values from each model and treated them as observed data (pseudo-observed datasets). We applied our pipeline to these datasets to verify our ability to successfully recover the true parameter values of each pseudo-observed dataset in the 95% HPDI as reported by ABCtoolbox. In addition, we used our inference pipeline in 1,000 pseudo-observed datasets for each of the tested demographic models to calculate the ration of the posterior estimates and their true value.

Likewise, we compared our pipeline and results to the methodology described in Wollstein *et al*. in 2010 to account for ascertainment bias using ABC^13^. Firstly, they simulated a demographic model involving the 3 HapMap populations and sampled a random number of haploid samples from each population in order to find polymorphic sites within regions of the ENCODE project data^23^. The physical distance distribution of these sites was then fitted to the observed distribution of the Affy 6.0 platform and, with ABC, the parameters of the previously mentioned demographic model as well as the number of ascertained samples were inferred. The summary statistics used were: observed heterozygosity, number of distinct haplotypes, number of the most frequent haplotype per population and the joint frequency spectrum. Secondly, the resulting posterior distribution of the demographic parameters and samples sizes were utilized as the prior for the consecutive investigated models involving the different Oceanian populations. Herein, we refer to this series of steps as Wollstein’s method.

We followed Wollstein’s steps to infer the ascertainment and demographic parameters of the Out-of-Africa and Mexican admixture models using our observed data. We reduced our summary statistics in order to replicate the set used in^13^: mean number of segregating sites, mean FST, mean number of different haplotypes, mean number of the most frequent haplotype in both the 10 kb loci and array data. We also retained the same percentage of simulations as in the aforementioned paper (0.02% of the total number of simulations).

We finally compared the mode and the width of the 95% HPDI of the parameters’ estimations obtained by the two methods and verified if the true values of pseudo-observed datasets were recovered by Wollstein’s method. The complete description of the validation and comparison of the pipeline can be found in the Supplemental Material.

## Results

### Results of the Out-of-Africa model

**Figure 4:**
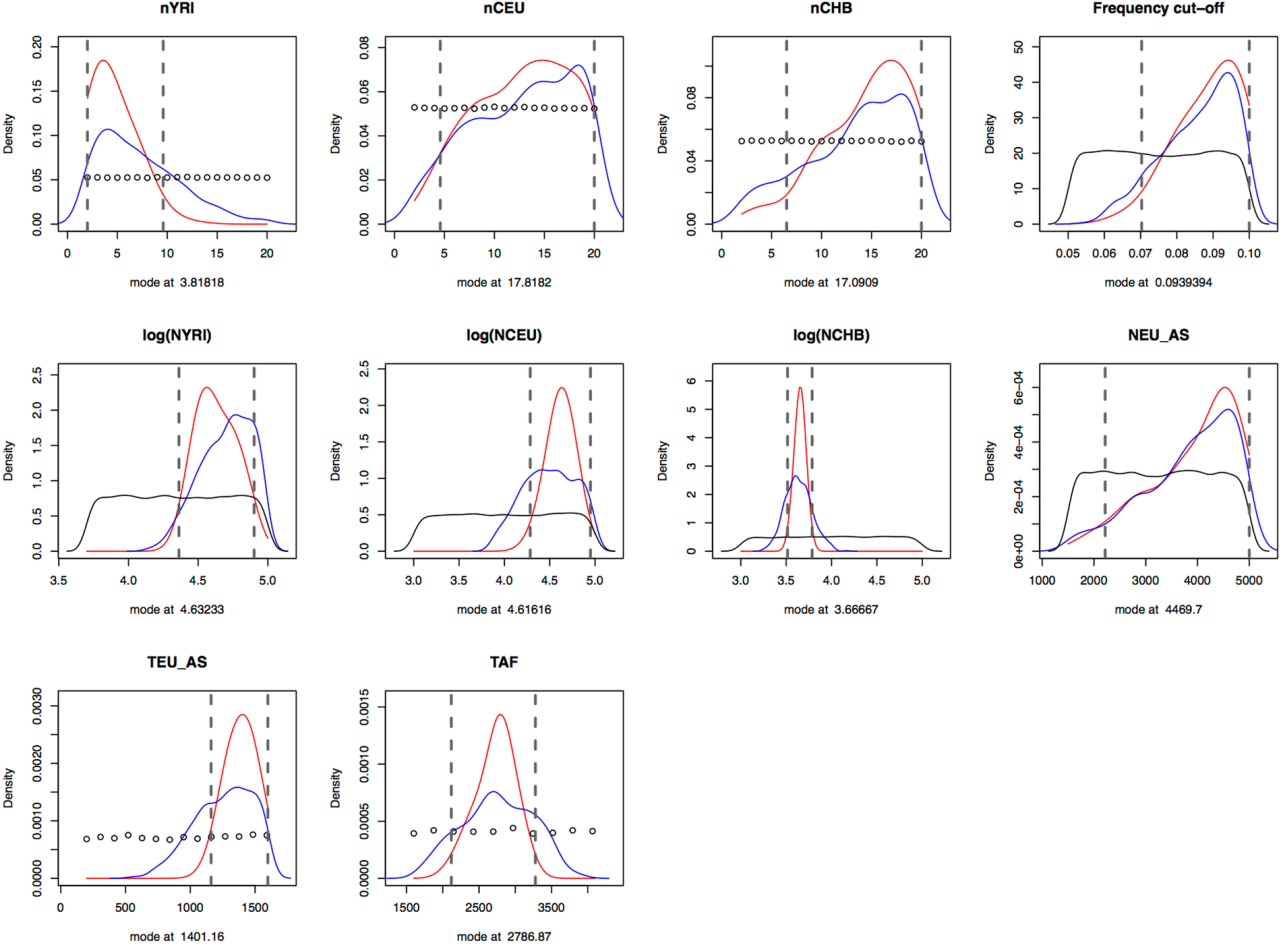
Posterior distributions of the discovery set and demographic parameters as estimated from the Out-of-Africa model using the observed data. The black curve corresponds to the prior distribution of the parameter values, while the blue one is the truncated prior distribution. The truncated prior distribution is built based on the parameter values of the retained simulations that are kept for analysis based on euclidean distance of the simulated summary statistics from the observed summary statistics, thus making these values a subset of the prior distribution and directly affecting the amount of information given for inference. The red curve is the posterior distribution, and the dashed lines are the 95% High Posterior Density Interval. Results estimated with 8 PLS components and 500 retained simulations.

We applied the inference pipeline to the Out-of-Africa model and obtained posterior estimates of the discovery set and demographic parameters (Table 1, Fig. 4). We inferred that 3.8 haploid samples came from YRI (95% HPDI.: 2–9.6), 17.8 from CEU (95% HPDI.: 4.58–20), and 17.1 from CHB (95% HPDI.: 6.5–20) to define the SNPs present in the Axiom array, with a frequency cut-off of 9.4% (95% HPDI.: 7–10%).

Moreover, we estimated that African and Eurasians populations diverged 69.7 kya (thousand years ago, 95% HPDI.: 52.9–81.9 kya), and that the time of divergence between CHB and CEU was 35.0 kya (95% HPDI.: 29.0–35.9 kya). We also found particularly large effective populations sizes for YRI (42.7 K, 95% HPDI.: 22.9–79.4 K) and CEU (41.7 K, 95% HPDI.: 19.1–79.4 K); whereas we estimated 4.7 K (95% HPDI.: 3.3–6.2 K) for CHB, and the size of the ancestral population of Eurasians was 4.5 K (95% HPDI.: 2.2–5 K).

### Results of the Mexican admixture model

We first confirmed that the MXL samples show a signal of admixture with the IBS and NXP populations as sources. In the PCA plot, the IBS and NXP samples formed tight clusters on opposite sides of the plot and the MXL individuals are found between these two clusters (Supplementary Fig. S3). The mean European contribution to the MXL individuals was 48.6% (sd 17.2%) according to the ADMIXTURE results (Supplementary Fig. S4).

**Figure 5:**
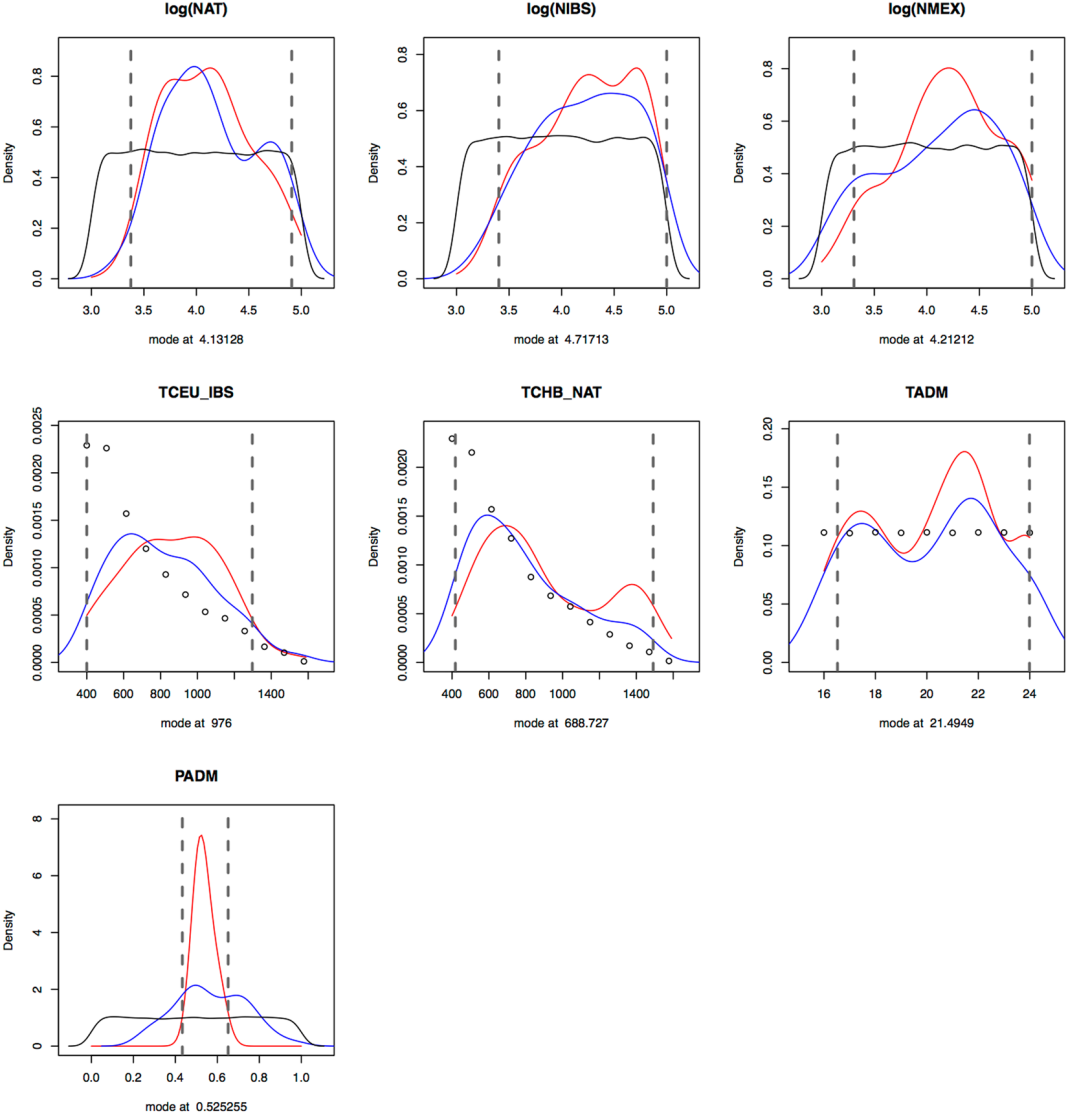
Posterior distributions of the demographic parameters of the Mexican admixture model. These posterior distributions correspond to the demographic parameters pertaining only to the Mexican, Iberian and Nahuas populations (see Fig. 3). Same color legend as Fig. 4. The posterior distributions of all the ascertainment and demographic parameters can be found in Supplementary Fig. S15. Results estimated with 8 PLS components and 100 retained simulations.

The posterior estimates are reported in Table 2 and the posterior distributions of the Mexican admixture specific parameters are shown in Fig. 5. We estimated the following discovery set of the Affy 6.0 array: 6.9 YRI haploid samples (95% HPDI: 2–17.4), 16 haploid samples (95% HPDI: 3.6–19.9); 7.5 haploid samples (95% HPDI: 2–17.6); and a frequency cut-off value of 6.1% (95% HPDI: 5.1–8.5%). The estimated time for the Out-of-Africa migration was 48.8 kya (95% HPDI: 40–62.3 kya), and the time of divergence between Europeans and Asians was 35.3 kya (95% HPDI: 26.4–39.9 kya). The effective populations sizes of these populations were 27.5 K (95% HPDI: 12.6–97.7 K), 12.3 K (95% HPDI: 5–43.7 K) and 3.5 K (95% HPDI: 1.8–4.8 K) individuals for YRI, CEU and CHB respectively. The size of the ancestral population to Europeans and Asians was estimated to be 4.1 K individuals (95% HPDI: 2.2–4.9 K).

Of special interest are the estimations for the IBS, NXP and MXL populations. The divergence time between Northern/Western Europeans and Iberian populations was 24.4 kya (95% HPDI: 10–32.4 kya), while the split between Han Chinese and the indigenous Mexican population was dated to be 17.2 kya (95% HPDI: 10.5–37.3 kya). The estimates of the effective population sizes of the Nahuas and Iberian populations were 13.5 K (95% HPDI: 2.4–81.3 K) and 52.5 K (95% HPDI: 2.5–100 K) respectively; while the Mexican Mestizo population size was 16.2 K (95% HPDI: 2–100 K). Moreover, the time of admixture between the Iberian and Nahua populations was estimated to have occurred almost 22 generations ago, or 550 years ago (95% HPDI: 413–600 ya); with the Iberian population contributing 53% (95% HPDI.: 43–65%) of the genomes.

### Results of the validation and comparison with other methods

The estimations of the parameter values of the pseudo-observed datasets were always within the 95% HPDI and the true values were most of the time recovered by the mode of the posterior distributions. Supplementary Figs S7 and S8 show the results of one pseudo-observed dataset from the Out-of-Africa model, and one pseudo-observed dataset from the Mexican admixture model.

We calculated the ratio of our posterior estimate and the true value of the parameters for 1,000 pseudo-observed datasets. For both tested models, the mode of the distribution of this ratio peaks close to the expected value of one and most of the obtained distributions peak at this value (Supplementary Figs S9 and S10). Yet, in the Mexican admixture model, the estimated values of the effective population sizes of Iberians, Mexican and Nahuas are smaller than their true values as the ratios are closer to 0.5; indicating that the posterior estimates were roughly half of the true value. This observation, however, is in agreement with our results when using the observed data of the Mexican admixture model.

We also compared the posterior estimates of the parameters that were obtained with our method and with Wollstein’s. The estimates of the ascertainment parameters are different, except for the frequency cut-off; and the 95% HPDI’s tend to overlap (Supplementary Table S3). Yet, we observed greater differences in the demographic parameters. The inferred effective populations sizes of the HapMap populations are larger when utilizing the Wollstein method, the 95% HPDI do not overlap, and the times of divergence are considerably recent: ~20 kya for the time of split between Eurasians, and ~47 kya for the Out of Africa migration. Also, some of Wollstein’s posterior distributions peak at the edge of the distributions like the frequency cut-off, the effective population size of Europeans and the time of split of Eurasians; and the mode of the truncated prior and posterior distributions do not coincide (Supplementary Fig. S11). The width of the 95% HPDI for seven parameters of the pseudo-observed data was smaller when using our method than Wollstein’s (Supplementary Table S3) and all intervals overlapped (Supplementary Fig. S12). However, neither method recovered the true value of the number of ascertained samples in Asians (nCHB), probably because its true value laid at the end of the prior distribution.

Regarding the Mexican admixture model, we first estimated the ascertainment and demographic parameters of the Out-of-Africa part of the model using the 10 kb loci and the Affy 6.0 data. We estimated that the number of haploid samples to define SNPs for the Affy 6.0 array were 19 from YRI, 8 from CEU and 11 from CHB. We then proceeded to use these posterior distributions as priors to simulate the IBS, NXP and MXL samples (Supplementary Fig. S13), following the topology of the admixture model and inferred the demographic parameters. The estimated population sizes and the admixture-related parameters are not radically different between the two methods, but the time of divergence between CEU and IBS and the time of divergence between CHB and NXP as estimated by Wollstein’s method are particularly old; 39.8 kya and 25 kya, respectively (Supplementary Table S5). Additionally, we noted that only two HPDIs inferred by our method were smaller than Wollstein’s, and that the posterior distributions of the effective population size of Iberians and the time of split between IBS and CEU have their mode right at the boundary of the distributions (Supplementary Fig. S13b).

## Discussion

One of the motivations to perform this work was the inability to know exactly the ascertainment strategy used to define SNPs during the creation of HapMap. Different papers have pointed out that HapMap utilized different SNP ascertainment procedures and so, the final ascertainment will be a mixture of ascertainment schemes. In 2005, Clark and colleagues showed the effect of ascertainment bias on the value of some summary statistics for the HapMap and Perlegen SNP datasets^4^. They were able to assemble enough information to obtain the possible distribution of the sample size (depth) in the discovery sample for HapMap and Perlegen. According to their results, between 5 and 10 samples were used in HapMap while 20 samples were used in Perlegen. Our estimates of the number of haploid samples used to find SNPs oscillate between 3 and 17, which may likely reflect the mixture of ascertainment strategies (See Tables 1 and 2).

Our estimates of the dispersal out of Africa and the divergence times between Eurasians are compatible with previous analyses, using mitochondrial DNA^24^, resequencing data^19,25^, and whole-genome information^20^. In contrast, our estimates of the effective population sizes seem to be elevated (Table 1). However, as no significant bias in the estimation of these parameters was observed in our validation, the elevated effective population sizes may reflect model misspecification.

An important difference between the previously mentioned publications and our Out-of-Africa model is the exclusion of migration and exponential growth in the studied populations. These demographic events will have an effect on the amount of genetic diversity of those populations. Nevertheless, the complexity of a demographic model increases the complexity of the parameter estimation with ABC, and for this reason, we decided to utilize a model with the smallest number of parameters that produced summary statistics with similar values to the observed summary statistics (Supplementary Fig. S2).

It is also very important to note that we included instantaneous growth in Africa (see Table 1, Figs 2 and 3) to ensure that the range of the simulated summaries encompassed the observed values. However, our pipeline did not have to power to accurately estimate this parameter (see Supplementary Fig. S16), and therefore we excluded this parameter from the inference process for both the Out-of-Africa and the Mexican model. This could potentially be explained by the type and amount of data used in our analysis as it has been previously noted that, to accurately infer the rate and time of growth in African populations, a large amount of data is required^26,27^.

The present-day Mexican population primarily descends from an admixture process between the indigenous resident populations and Spanish explorers that entered Mexico after 1492. A minor source of current genetic variation in Mexico traces to African slaves that were brought to the Americas^17,28^; however, their contribution was not considered here.

We chose to examine the case of admixture in Mexico for two reasons: (1) at the time of this work only SNP genotypes from indigenous Mexican populations were publicly available^17^, and (2) the effects of ascertainment bias are expected to be stronger given the divergence between indigenous Mexican populations and those that were part of the SNP discovery panels (i.e., HapMap populations). The choice of the Nahuas from Puebla as a representative of the indigenous population was motivated by its location in central Mexico and the number of available samples. We also made the assumption that MXL individuals were Mestizo individuals with predominately more ancestry from central groups which held a large territory, as opposed to more isolated and small populations (e.g. Mayans).

Regarding the admixture dynamics of this historical encounter, our results indicate an admixture time of 21.5 generations ago (538 years ago, 95% HPDI: 413–600 ya), in agreement with recorded history and with previous estimates of admixture timing using alternative approaches such as ancestry block length distribution^28–31^. When an admixture event takes place, the chromosomes that are brought together are shuffled by recombination during meiosis. As the time since admixture increases, chromosomes from the admixed population become mosaics of shorter segments of DNA from the sources populations. The ancestry switch points are inherited like point mutations and can be used as markers to define ancestry blocks or haploblocks.

In contrast with such methods, our approach enables bias-free inference of additional demographic parameters, like the effective population sizes and times of divergence which have been typically inferred from sequencing data only until now. Our inference of 53% Spanish contribution to the Mexican Mestizo genetic pool (95% C.I.: 43–65%) is consistent with other published estimates for Mexican Americans (48%,^17,19,29,31^). The small difference in the estimates could be due to the fact that the MXL samples were collected outside of Mexico and probably experienced more recent gene flow from Europeans in the U.S.^30^. The estimate of the divergence between CHB and NXP (18 kya) corresponds to the estimated time of crossing of the Bering land bridge during the Last Glacial Maximum by the ancestors of Americans^32–34^. However, we inferred a very old split time between Northern/Western European and Iberian populations (24 kya), as opposed to previous results that point out to a much recent divergent time between European populations (e.g. divergence time between Dutch and Central Italian: 7 kya^25^,) and the high degree of relatedness in those populations^35^.

We estimated similar population sizes for the IBS, NXP and MXL populations (10,000) which are lower estimates compared to previous results using data generated by 1000 Genomes Project^34^. In contrast, using runs of homozygosity, the effective population size of the Nahua population was calculated to be around 3,100 individuals^17^. These large differences in the estimates could be due to the particular dataset analyzed, the complexity and assumptions of the models employed, and/or due to the fact that our method has little power to estimate these parameters (see the width of the HPDIs in Table 2).

In general, the results of the validation of our pipeline show that it is possible to estimate without bias demographic and ascertainment parameters (See Supplementary Figs S9 and S10). When compared to Wollstein’s method, we found that our pipeline provided more accurate estimates of the parameter values, even though the 95% HPDI’s almost always overlapped between the two approaches. In some cases, the disparity between the parameter estimates were of an order of magnitude (see effective population sizes and times of divergence in Supplementary Table S3).

The differences in the estimates between our pipeline and Wollstein’s method could be explained by the fact that we implemented the analyses with the same proportion of simulations that Wollstein seemed to have retained (0.02% of the total), which probably provided a small amount of information to ABCtoolbox for the inference. Another important difference is the estimation of the ascertainment and demographic parameters of models at the same time and the incorporation of bootstrap sampling to have information of the joint distribution of the parameters.

In sum, we showed that our developed pipeline enables to do demographic inference using SNP array data ABC, can be applied to data from different commercial arrays and that the only requirement for its application is to have whole genome information of the populations used to define SNPs and the SNP array data of the populations of interest.

## Electronic supplementary material

Dataset S1

Dataset S2

Dataset S3

Dataset S4

Supplementary material
