# Supplementary material for: Modeling SNP array ascertainment with Approximate Bayesian Computation for demographic inference
Source: Sci Rep. 2018 Jul 5;8:10209. doi: 10.1038/s41598-018-28539-y (PMC6033855; doi:10.1038/s41598-018-28539-y)
Supplement: Supplementary file 5 — Supplementary material [file 41598_2018_28539_MOESM5_ESM.pdf]

Supplemental Methods

Modeling SNP array ascertainment with Approximate Bayesian Computation for demographic inference

Consuelo D. Quinto-Cortés[1,*], August E. Woerner[2], Joseph C. Watkins[3] and Michael F. Hammer[4,*]

[1]National Laboratory of Genomics for Biodiversity (LANGEBIO), CINVESTAV, Irapuato, 36821, Mexico
[2]Center for Human Identification, University of North Texas Health Science Center, Texas, 76107, U.S.A.
[3]Department of Mathematics, University of Arizona, Tucson, Arizona, 85721, U.S.A.
[4]ARL Division of Biotechnology, University of Arizona, Tucson, Arizona, 85721, U.S.A.
[*]email: mfh@email.arizona.edu, consuelo.quinto@cinvestav.mx

Selection of neutral genomic regions

To infer the ascertainment of the Axiom and Affy 6.0 arrays, we first determined a set of neutral genomic regions using the Neutral Regions Explorer[1] as to reduce the potential interference of natural selection in demographic inference. Several criteria were applied such as the exclusion of genes, segmental duplications and other repeats, copy number variants, and an interval of 0.1 cM between non-overlapping regions. We restricted the length of this preliminary set of regions to 10 kb. These given regions were extracted from the CGI genomes, and any site that was not genotyped correctly in at least one of the sampled individuals was removed from subsequent analyses. Additionally, we extracted sites from the CGI genomes with the same physical positions as the SNPs present in the Axiom array. The final working of set of regions comprised 1386 neutral genomic regions, referred herein as '10kb loci'. We calculated the number of segregating sites in the each of the 10kb loci as well as the number of SNPs that are present in the Axiom array that are segregating in the YRI, CEU and CHB samples (Supplementary Fig. S1).

Coalescent simulations and summary statistics

For all the simulations performed in this work, we used a customized version of the simulator MaCS (Markovian Coalescent Simulator)[2] for Python which allows faster processing of the coalescent simulations. We used a mutation rate of 2.5e-8 mutations per site[3], a per nucleotide recombination rate of 1e-8, and the HapMap genetic map[4].

We set up the simulations in the following way: random values for the ascertainment and demographic parameters' prior distributions were chosen and used to simulate the whole set of 1386 10kb loci. The number of samples simulated corresponded to the number of real samples from the studied populations (Model 1: YRI, CEU, CHB; Model 2: YRI, CEU, CHB, NXP, IBS, MXL; See Supplementary Table S1) plus the number of haploid samples in the discovery panel (drawn from the prior distribution). One simulation includes: the simulation of the whole genome data (10kb loci) in the sample that includes 9 YRI, 9 CEU and 4 CHB diploid individuals, the simulation of the ascertainment sample (referred as discovery panel in the manuscript) and the ascertained data (ascertained SNPs in each of the 10kb loci in the discovery panel).

For each locus in the 10kb loci, we recreated the number and the physical distribution of the SNPs found in the real array, and computed summary statistics in those SNPS. We also calculated the same summary statistics in the whole-genome data (all SNPs in the 10kb region). For one simulation, we calculated the mean and standard deviations of all the summary statistics (from whole-genome and ascertained data) across the 1386 10kb loci (Out-of-Africa model: 108 summary statistics; Mexican admixture model: 96 summary statistics). These values were later transformed into PLS components.

The summary statistics used in this work were: the number of segregating sites, singletons, doubletons, Tajima's D, number of distinct haplotypes and number of the most frequent haplotype per population, F_{ST} and number of shared and private haplotypes between pairs of populations. These statistics were later transformed into Partial Least Squares (PLS) components using the 'transform' option of ABCtoolbox[5]. We calculated the weight of each of the summary statistics when the PLS components were computed. The contribution of the top 6 summary statistics for each of the models are shown in Supplementary Table S2. The full list of the contributions as well as the weights of each of the PLS components can be found in the supplementary data files S1-S4.

PLS produce orthogonal and independent combinations from the simulated summary statistics (predictor variables) and they eliminate any obvious correlations between the statistics. An important characteristic of PLS components is that each of them explains the variability of the demographic parameters (the response variables) by maximizing the covariance matrix of the predictor and response variables[6,7].

Estimation optimization and inference

Before any inference was done, we verified that the explored parameter priors in the one million simulations of the Out-of-Africa model did generate summary statistics similar to the observed in the YRI, CEU and CHB samples (Supplementary Fig. S2).

We inferred the ascertainment and demographic parameters of this model with the software ABCtoolbox[5]; which applies a General Linear Model regression adjustment to estimate the marginal posterior densities of parameters[8].

It is usual practice in ABC to generate a large number of simulations to make sure that the parameter space set by the prior distributions is being covered (almost) entirely. However, only a subset of the total simulations are used in the actual inference (between 0.1% and 1%) and the retained simulations are determined by the Euclidean distance between the observed and simulated summaries. Another important variable that needs to be taken into account is the number of summary statistics (in our case, PLS components) that are used in an ABC framework. The inclusion of too few PLS components might not provide enough information, while too many components may overfit the model. The number of simulations that are included in the analysis can have the same effect.

For this reason, and in order to optimize the estimation of the parameters values, we used ABCtoolbox with different combinations of the number of PLS components and of the number of retained simulations. We used 100, 500, 1000, 2000 and 5000 retained simulations and a range of 2-15, 20, 30 and 50 PLS components. For each combination of these two variables, we re-sampled the one million simulations with replacement to obtain a joint distribution of the parameter estimates. We later computed the variance-covariance matrix of the posterior estimates of the parameters (to maintain the structure of the joint estimation of the parameters), and generated 1000 random samples from a multivariate normal distribution. We then performed simulations with those values, standardized the observed and simulated summary statistics and calculated the Euclidean distance between these values. Lastly, we selected the combination of PLS components and number of retained simulations with the smallest Euclidean distance, confirmed that its parameter values generated summary statistics similar to the observed ones (with a principal component analysis), and obtained the posterior mode and 95% High Posterior Density Intervals (HPDI) for each parameter.

Mexican admixture analysis

We merged the Affy 6.0 genotypes from all six populations with PLINK[9] and sites with missing data were removed as well as first-degree relatives. The final merged dataset contained 594,236 SNPs in 310 individuals.

We performed a principal components analysis with EIGENSTRAT[10] and used the ADMIXTURE software[11] with only the IBS, MXL and NXP samples to determine ancestry proportions. MXL individuals with more than 99% European or 99% Indigenous ancestry were excluded from the merged set, as they were not considered to be representatives of the admixture process (Supplementary Fig. S3). For these two analyses, SNPs in linkage disequilibrium were pruned with PLINK[9] (–indep-pairwise 50 5 0.8). In addition, NXP samples that showed more than 99% European ancestry were also filtered out in order to limit the amount of non-Native American contribution in this source population. Since only eleven NXP individuals were retained, the IBS and MXL populations were subsampled to avoid sample size bias and to decrease the computation time of the coalescent simulations. After we reduced the number of samples, we verified with ADMIXTURE that the admixed individuals did not have extreme values of European or indigenous ancestry (mean European ancestry=45.9%, Supplementary Fig. S4).

As with the simulations of the Out-of-Africa model, we performed a principal components analysis on the observed and simulated data from this demographic model and corroborated that the explored parameter spaces generated summary statistics similar to the observed ones (Supplementary Fig. S5).

Validation of the pipeline

To verify the proposed inference pipeline, we performed two sets of analyses. Firstly, for the Out-of-Africa model, we estimated demographic parameters using exclusively the information from the 10kb loci and compared the obtained values to the estimates we obtained with the pipeline that accounts for ascertainment bias. The resulting posterior estimates are similar to the ones obtained applying our pipeline (Supplementary Table S3, Supplementary Fig. S6).

Secondly, we took 1000 random pseudo-observed datasets (one from each of the two models considered in this study) in order to examine the power of the pipeline to correctly recover the true parameter values. Our estimations were always within the 95% HPDI and the true values were most of the time recovered by the mode of the posterior distributions. Supplementary Figures S7 and S8 show the results of one pseudo-observed dataset from the Out-of-Africa model, and one pseudo-observed dataset from the Mexican admixture model.

In addition, for each of the 1000 pseudo-observed datasets, we calculated the ratio of the estimated parameters and their true value. If these two values are similar, then the ratio should be close to one. For this analysis, we used the combination of PLS components and retained simulations that provided the best estimates for the observed data in each case. For both tested models, for almost all the parameters, the mode of distributions of the ratio is close to the expected value of one (Supplementary Figures S9 and S10). However, in the case of the Log(NAT), Log(NIBS) and Log(NMEX) of the Mexican admixture model, the estimated values were lower than their true values and the ratios are closer to 0.5; indicating that the estimated values were roughly half of the true value. This observation is consistent with the estimates we obtained for the same parameters when we used the real data.

Comparison with previous published methods

In 2010, Wollstein and colleagues investigated the demographic history of Oceania with SNP microarray data (Affy 6.0) and described a two-step approach to account for ascertainment bias using ABC[12]. Our pipeline substantially differs from Wollstein's method in the following aspects: a) we find SNPs based on a discovery panel and a frequency cut-off vs. finding all variable sites; b) we use a large number of summary statistics (that are later linearly transformed) vs. a small number of statistics and no linear transformation; c) we search for the optimal number of retained simulations and summary statistics for parameter inferences vs. just retaining a small percentage of simulations for inference; d) we utilize a bootstrap strategy vs. no bootstrap; and e) we perform the inference of ascertainment and demographic parameters at the same time vs. a two-step process.

We implemented Wollstein's method following the description provided in the supplementary materials of the paper as close as possible so some of the observed disparities could be justified by our execution of his approach and the obvious changes on the data and models utilized.

We followed Wollstein's steps to infer the ascertainment and demographic parameters of the Out-of-Africa and Mexican admixture models using our observed data and pseudo-observed datasets. We reduced our summary statistics in order to replicate the set used in[12]: mean number of segregating sites, mean F_{ST}, mean number of different haplotypes, mean number of the most frequent haplotype in both the 10kb loci and array data. We also retained the same percentage of simulations as in the aforementioned paper (˜0.02% of the total number of simulations). We then compared the mode and the width of the 95% HPDI of the parameters' estimations obtained by the two methods and verified if the true values of pseudo-observed datasets were recovered by Wollstein's method.

We also verified if the truncated prior distribution was informative to compute the posterior distribution. This distribution is built based on the parameter values of the retained simulations that are kept for analysis, thus making these values a subset of the prior distribution and directly affecting the amount of information given for inference. We observed some issues in the performance of Wollstein's method to estimate appropriately some of the parameters, using real and simulated data. As seen in Supplementary Figures S11 and S12, the truncated prior distributions were not informative (looking alike the prior distribution) in some cases or the mode did not coincide with the posterior mode.

We used nine pseudo-observed datasets of the Mexican admixture model to examine the general performance of Wolllstein's method (Supplementary Table S7). In each cell of this table, it is indicated whether or not the true value of the parameters was recovered by the 95% HPDI calculated by the two methodologies. There was not a obvious trend about what parameters could not be estimated properly. Wollstein's method failed 46% of the time (29/63) to recover the true values, while our method failed 8% (5/63) of the time. Supplementary Table S8 shows the results of only one of these sets. For almost half of the parameters, our 95% intervals were smaller than Wollstein's and they overlapped, with the exception of the time of split between CEU and IBS (Supplementary Figures S13 and S14).

Since we implemented the analyses with the same proportion of simulations that Wollstein seemed to have retained (0.02% of the total), the observed differences could be explained by the small amount of information that was given to ABCtoolbox for the inference. Additionally, both this work and[12] used genotype data derived from the Affy 6.0 array and inferred the ascertainment scheme for the SNPs present in this array. The estimates are strikingly different: 19 haploid samples from YRI vs. 2, 8 vs. 1 from CEU, and 11 vs. 2 from CHB (see Supplementary Figure S13).

Scripts

The list of the 10kb loci and all the scripts used in this work can be found in the following link:
`https://bitbucket.org/cdquinto/ascertainment-bias-scripts/`

Supplementary data files

Dataset S1: Contribution of the summary statistics to the PLS transformation of the Out-of-Africa model analysis.
Dataset S2: Contribution of the summary statistics to the PLS transformation of the Mexican admixture model analysis.
Dataset S3: Value of each of the PLS components of the Out-of-Africa model analysis.
Dataset S4: Value of each of the PLS components of the Mexican admixture model.

The abbreviations used in those files correspond to :
Populations: Af - Africa; Eu - Europe; As - Asia; Mex - Mexicans; Ibe - Iberians; Nat - Nahuas.
Genetic summaries: SegS - segregating sites; Sing - singletons; Dupl - doubletons; TajD - Tajima's D; FST - Wright's fixation index; Pi - nucleotide diversity; hap - haplotypes.
Standard summaries: Nb - number; m - mean; sd - standard deviation; CGI - genomic summaries; ASC - summaries from ascertained SNPs.

Supplemental Tables and Figures

Supplementary Table S1. **Summary of the data included in this study**

Population	PopID	Initial n	Final n	Region	Data	Reference
Yoruba	YRI	9	9	West Africa	Complete Genomics<br>Affymetrix Axiom Genome-Wide Human Array<br>Affymetrix 6.0	13<br>14
European American	CEU	9	9	Northern and Western Europe	Complete Genomics<br>Affymetrix Axiom Genome-Wide Human Array<br>Affymetrix 6.0	13<br>14
Han Chinese	CHB	4	4	Beijing, China	Complete Genomics<br>Affymetrix Axiom Genome-Wide Human Array<br>Affymetrix 6.0	13<br>14
Spanish	IBS	162	11	Southern Europe	Affymetrix 6.0	15
Mexican American	MXL	104	11	California, USA	Affymetrix 6.0	15
Nahua	NXP	22	11	Puebla, Central-East Mexico	Affymetrix 6.0	15

Supplementary Table S2. Contributions of summary statistics

Out of Africa model	Weight	Mexican admixture model	Weight
Nb different haplotypes in Africa (ASC,sd)	2.80145043	Nb doubletons in Asia (CGI,m)	2.02944394
Tajima's D in Europe (CGI,m)	2.45108901	Nb shared haplotypes Iberian-Mexicans (ASC,sd)	2.02208214
Nb shared haplotypes Africa-Europe (ASC,sd)	2.40768415	Nb different haplotypes Mexicans (ASC,sd)	1.81347634
Nb segregating sites Europe (ASC,sd)	2.08818833	Tajima's D in Iberians (ASC,sd)	1.7469355
Nb shared haplotypes Europe-Asia (ASC,sd)	2.07461472	Mode haplotypes Mexicans (ASC,sd)	1.45501021
Nb segregating sites Africa (CGI,m)	1.64600266	Pi Nahuas (ASC,sd)	1.44987776

Abbreviations: Nb - number; CGI - genomic summaries; ASC - summaries from ascertained SNPs; m - mean; sd - standard deviation

Supplementary Table S3. **Comparison of estimates of the Out-of-Africa demographic parameters**

Parameter	Mode 10kb loci (95% HDPI)	Mode 10kb loci and pseudo-array (95% HPDI)
**log10(NYRI)**	4.65 (4.05-4.99)	4.63 (4.36-4.90)
**log10(NCEU)**	4.13 (3.78-4.46)	4.62 (4.28-4.95)
**log10(NCHB)**	3.53 (3.27-3.73)	3.67 (3.52-3.79)
**NEU_AS**	2772.73 (1588.38-4865.49)	4469.7 (2216.22-5000)
**TEU_AS**	1005.48 (426-1436.49)	1401.16 (1160.94-1599)
**TAF**	2307.07 (1612.63-3278.14)	2786.87 (2117.68-3277.96)

The first column contains the estimates of parameters using only summary statistics from the 10kb loci (free of ascertainment). The second column has the estimates obtained with our proposed inference pipeline. Parameter labels correspond to those given in Figure 1. Divergence times are given in generations units. For posterior distributions, see Figure S6.

Supplementary Table S4. Comparison of inferred posterior estimates of the parameters of the Out-of-Africa from observed data

Parameter	Estimated mode Our pipeline	95% HPDI Our pipeline	Width	Estimated mode Wollstein	95% HPDI Wollstein	Width
**nYRI**	3.82	2-9.59	7.59	6.55	3-13.2	10.2
**nCEU**	17.82	4.58-20	15.42	9.82	7.06-19.9	12.84
**nCHB**	17.09	6.5-20	13.5	6.55	2.72-13.18	10.46
**Frequency cut-off**	0.094	0.07-0.1	0.03	0.1	0.091-0.1	0.009
**log10(NYRI)**	4.63	4.36-4.9	0.54	4.88	4.7-5	0.3
**log10(NCEU)**	4.61	4.28-4.95	0.67	5	4.8-5	0.2
**log10(NCHB)**	3.67	3.52-3.79	0.27	4.11	3.96-4.25	0.29
**NEU_AS**	4469.7	2216.22-5000	2783.78	1500	1500-2244.15	744.15
**TEU_AS**	1401.16	1160.94-1599	438.06	878.3	687.53-1205.93	518.4
**TAF**	2786.87	2117.68-3277.96	1160.28	1877.78	1600-2224.7	624.7

See posterior distributions in Figure S11.

Supplementary Table S5. Comparison of inferred posterior estimates of the parameters of the Out-of-Africa model from simulated data

Parameter	True value	Estimated mode Our pipeline	95% HPDI Our pipeline	Width	Estimated mode Wollstein	95% HPDI Wollstein	Width
**nYRI**	12	10.91	4.69-16.7	12.01	5.82	3.32-16.2	12.88
**nCEU**	9	17.27	3.94-19.53	15.59	8	3.35-19.29	15.94
**nCHB**	20	14.73	5.11-19.58	14.47	8	2.72-19.51	16.79
**Frequency cut-off**	0.053	0.062	0.051-0.08	0.029	0.059	0.051-0.087	0.036
**log10(NYRI)**	4.12	4.34	3.9-4.89	0.99	4.25	3.98-4.78	0.8
**log10(NCEU)**	4.64	4.72	4.45-4.94	0.49	4.74	4.37-4.97	0.6
**log10(NCHB)**	4.20	4.21	3.92-4.54	0.62	4.15	3.92-4.4	0.48
**NEU_AS**	4477.0	4575.76	3656.34-4958.15	1301.81	4611.11	3685.15-4966.27	1281.12
**TEU_AS**	1311.0	1288.11	1123.82-1468.95	345.13	1330.51	924.12-1568.38	644.26
**TAF**	3624.0	3569.7	3340.48-3789.81	449.33	3594.95	3096.96-4024.94	927.98

Posterior mode and 95% HPDI of one pseudo-observed data set. We compared the true parameter values to the estimated mode and the width of the corresponding confidence intervals. See posterior distributions in Figure S12.

Supplementary Table S6. **Comparison of inferred posterior estimates of the parameters of the Mexican admixture model from observed data**

Parameter	Estimated mode Our pipeline	95% HPDI Our pipeline	Width	Estimated mode Wollstein	95% HPDI Wollstein	Width
**log10(NNXP)**	4.13	3.38-4.91	1.53	3.75	3.42-4	0.58
**log10(NIBS)**	4.71	3.4-4.99	1.59	5	4.65-5	0.35
**log10(NMXL)**	4.21	3.3-4.99	1.69	4.13	3.62-4.93	1.31
**TCEU_IBS**	976	400-1297	897	1592	1434.94-1592	157.06
**TCHB_NXP**	689	418-1492	1074	1007.09	456-1207	751
**TADM**	21.5	16.53-23.99	7.46	21.66	17.98-23.7	5.72
**PADM**	0.52	0.43-0.69	0.26	0.59	0.53-0.66	0.13

See posterior distributions in Figure S13.

Supplementary Table S7. Comparison of inferred posterior estimates of the parameters of the Mexican admixture model from simulated data sets

	In 95% HPDI? Our pipeline									In 95% HPDI? Wollstein's method								
**Parameter**	1	2	3	4	5	6	7	8	9	1	2	3	4	5	6	7	8	9
**log10(NAT)**	Y	Y	Y	Y	Y	Y	Y	Y	*N*	*N*	Y	*N*	*N*	*N*	Y	Y	Y	*N*
**log10(NIBS)**	Y	Y	Y	Y	Y	Y	Y	Y	Y	*N*	*N*	*N*	*N*	*N*	*N*	Y	Y	Y
**log10(NMEX)**	Y	Y	*N*	Y	Y	Y	*N*	*N*	Y	Y	Y	*N*	Y	Y	Y	*N*	Y	Y
**TCEU_IBS**	Y	Y	Y	Y	Y	Y	Y	Y	Y	*N*	Y	Y	*N*	Y	*N*	Y	*N*	*N*
**TCHB_NAT**	Y	Y	Y	Y	Y	Y	Y	Y	Y	*N*	*N*	Y	*N*	*N*	Y	*N*	*N*	Y
**TADM**	Y	Y	Y	Y	*N*	Y	Y	Y	Y	Y	*N*	Y	*N*	*N*	Y	Y	Y	Y
**PADM**	Y	Y	Y	Y	Y	Y	Y	Y	Y	Y	Y	Y	Y	Y	Y	*N*	*N*	Y

In each cell of the table it is indicated whether or not the true values from nine pseudo-observed datasets of the Mexican admixture model were recovered in the 95% HPDI calculated by the two methodologies.

Supplementary Table S8. Comparison of inferred posterior estimates of the parameters of the Mexican admixture model from simulated data

Parameter	True value	Estimated mode Our pipeline	95% HPDI our pipeline	Width	Estimated mode Wollstein	95% HPDI Wollstein	Width
**log10(NAT)**	3.53	3.65	3.29-4.54	1.25	3.44	3.11-3.83	0.72
**log10(NIBS)**	4.50	4.70	4.22-4.99	0.77	3.53	3.17-3.89	0.72
**log10(NMEX)**	3.74	4.52	3.28-5	1.72	4.60	3.14-4.99	1.85
**TCEU_IBS**	634	496.72	400-788.20	388.2	400	400-454.27	54.27
**TCHB_NAT**	661	533.11	400-825.96	425.96	445.82	400-701.55	301.55
**TADM**	17	16.48	16-21.83	5.83	20.28	16.70-23.64	6.94
**PADM**	0.89	0.89	0.79-0.98	0.19	0.79	0.67-0.90	0.23

Posterior mode and 95% HPDI of one pseudo-observed data set. We compared the true parameter values to the estimated mode and the width of the corresponding confidence intervals. See posterior distributions in Figure S14.

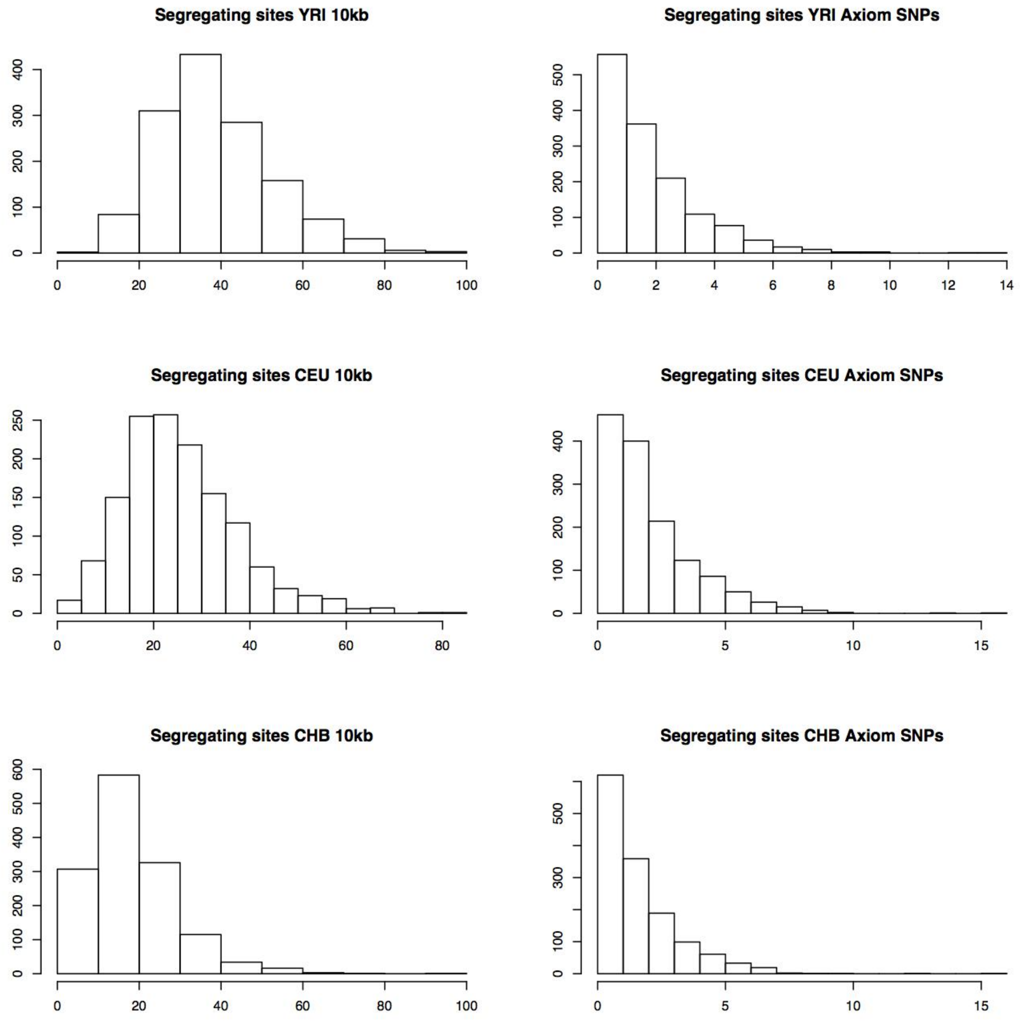

Supplementary Figure S1. Histograms with the distribution of segregating sites in the 10kb loci and of the Axiom SNPs. The distribution of segregating sites in the 10kb regions is depicted in the right panel. On the left, the histograms correspond to the number of segregating sites of the Axiom SNPs in the 10kb loci.

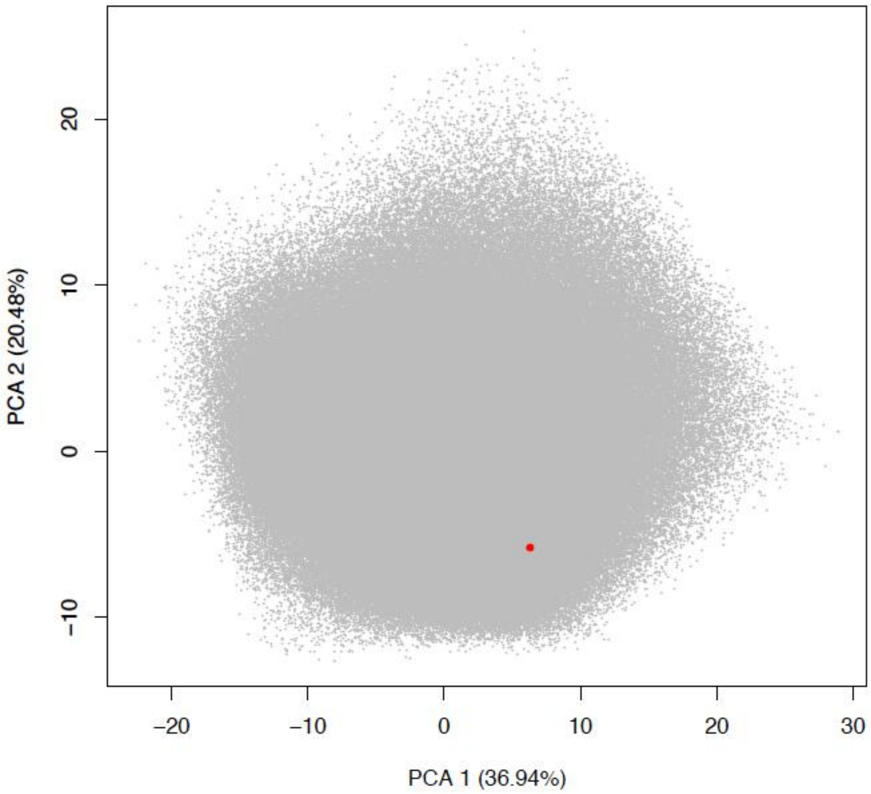

Supplementary Figure S2. Principal components analysis of the coalescent simulations of the Out-of-Africa model. The grey cloud represent the simulated summary statistics while the red dot corresponds to the observed summary statistics.

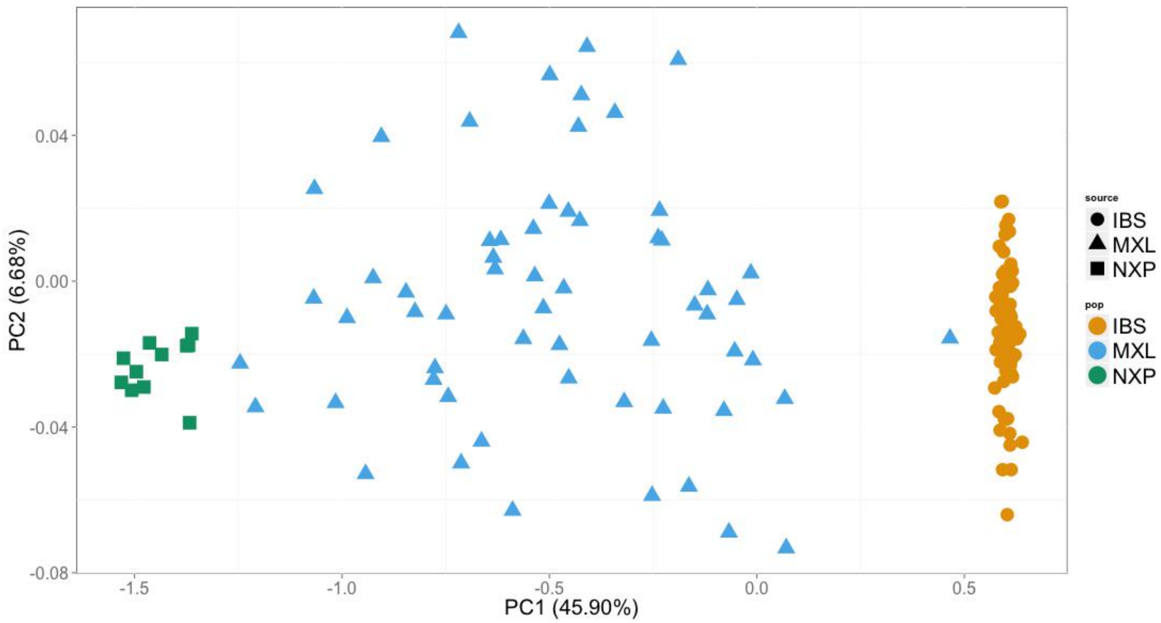

Supplementary Figure S3. Principal component analysis of the IBS, NXP and MXL individuals using genome-wide SNP data. PCA with all the unrelated MXL individuals from the 1000 Genomes Project. The orange points represent the individuals with Iberian ancestry (IBS), green points designate the Nahuas from Puebla (NXP); and light blue points correspond to the MXL samples. PCA with IBS, NXP and MXL samples with the two outliers removed. The analysis was done with 594, 236 SNPs in 151 individuals. The percentage of the variance explained by the first two principal components are between parenthesis.

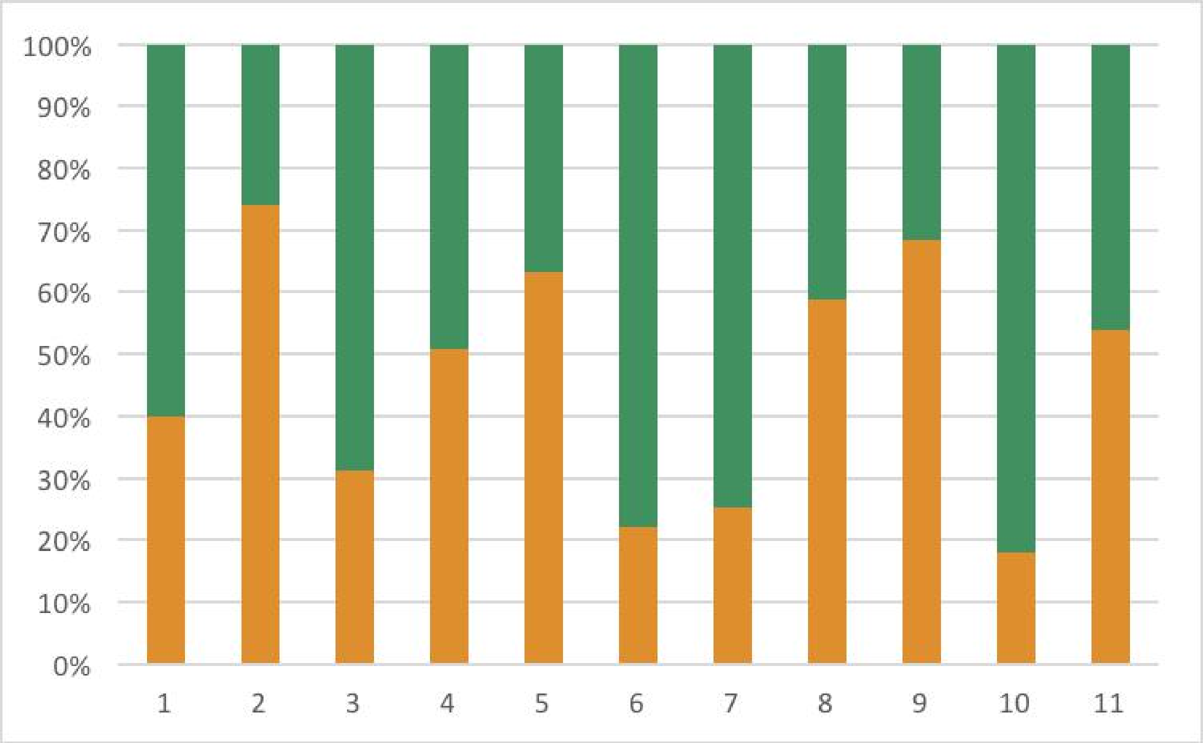

Supplementary Figure S4. Admixture plot of MXL individuals based on genome-wide SNP data. Ancestry proportions estimated in 11 Mexican individuals using ADMIXTURE[11] when the number of clusters was set to 2 (594, 236 SNPs). The orange color corresponds to the inferred Spanish ancestry, and the green one depicts the indigenous Native American contribution. These 11 individuals were used in the subsequent analysis. The average Iberian ancestry is 46%, and the indigenous contribution 54%.

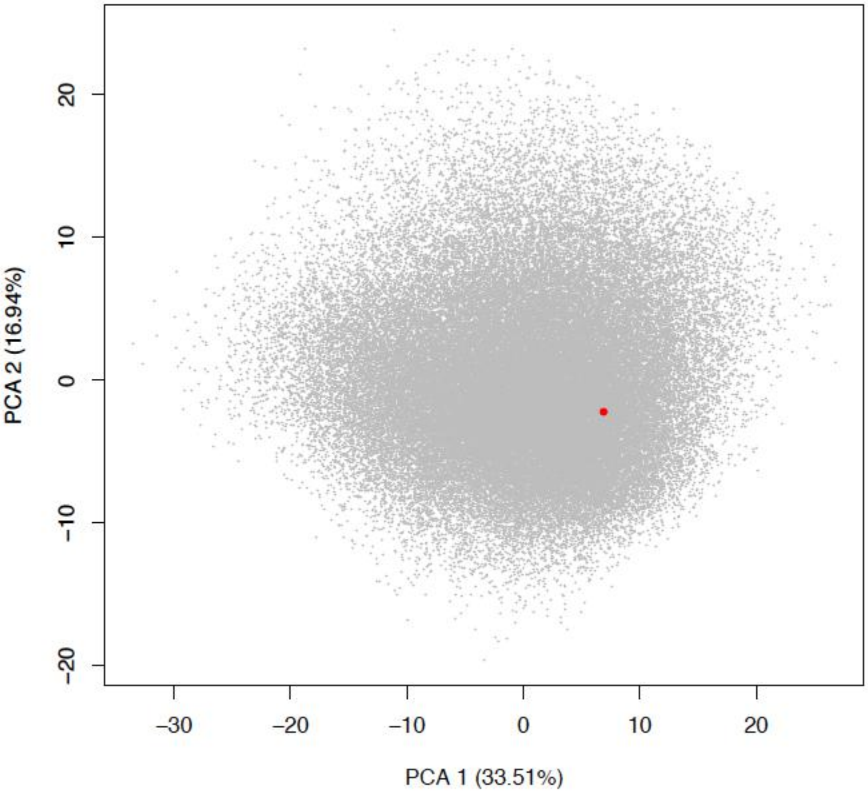

Supplementary Figure S5. Principal component analysis of the coalescent simulations of the Mexican admixture model. The grey cloud represent the simulated summary statistics while the red dot corresponds to the observed summary statistics.

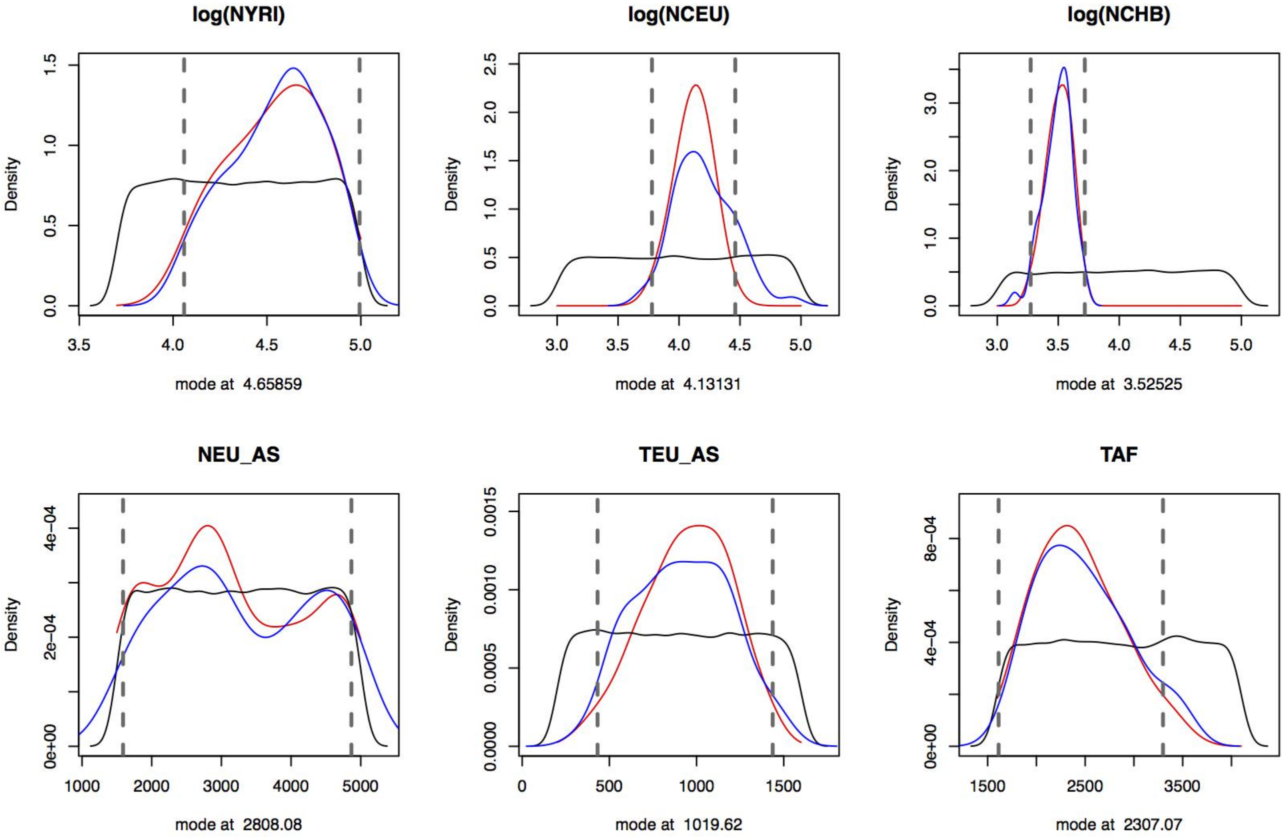

Supplementary Figure S6. Posterior distributions of the demographic parameters of the Out-of-Africa model based on the 10kb loci summaries only. SThe black curve corresponds to the prior distribution of the parameter values, while the blue one is the truncated prior distribution. The truncated prior distribution is the distribution of the parameters kept after the rejection process (part of ABCtoolbox's method). The red curve is the posterior distribution, and the dashed lines are the 95% High Posterior Density Interval.

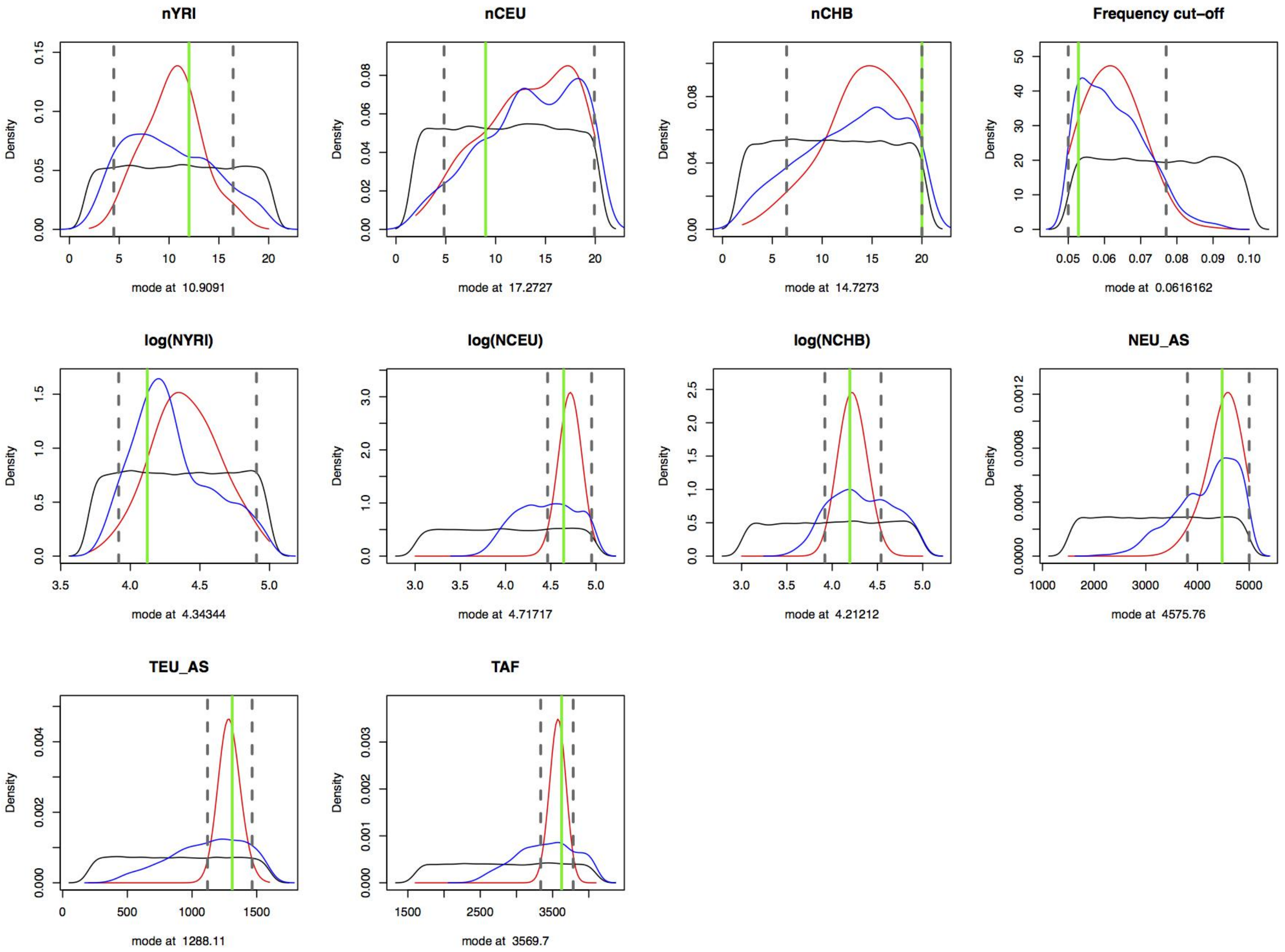

Supplementary Figure S7. Posterior distributions of the discovery set and demographic parameters as estimated from the Out-of-Africa model using one pseudo-observed data set. The black curve corresponds to the prior distribution of the parameter values, while the blue one is the truncated prior distribution. This distribution is built based on the parameter values of the retained simulations that are kept for analysis based on euclidean distance of the simulated summary statistics from the observed summary statistics, thus making these values a subset of the prior distribution and directly affecting the amount of information given for inference. The red curve is the posterior distribution, and the dashed lines are the 95% High Posterior Density Interval. The true values of the parameters (in bright green) were recovered by the mode of the posterior distributions (in almost all the cases) and by the 95% High Posterior Density Interval.

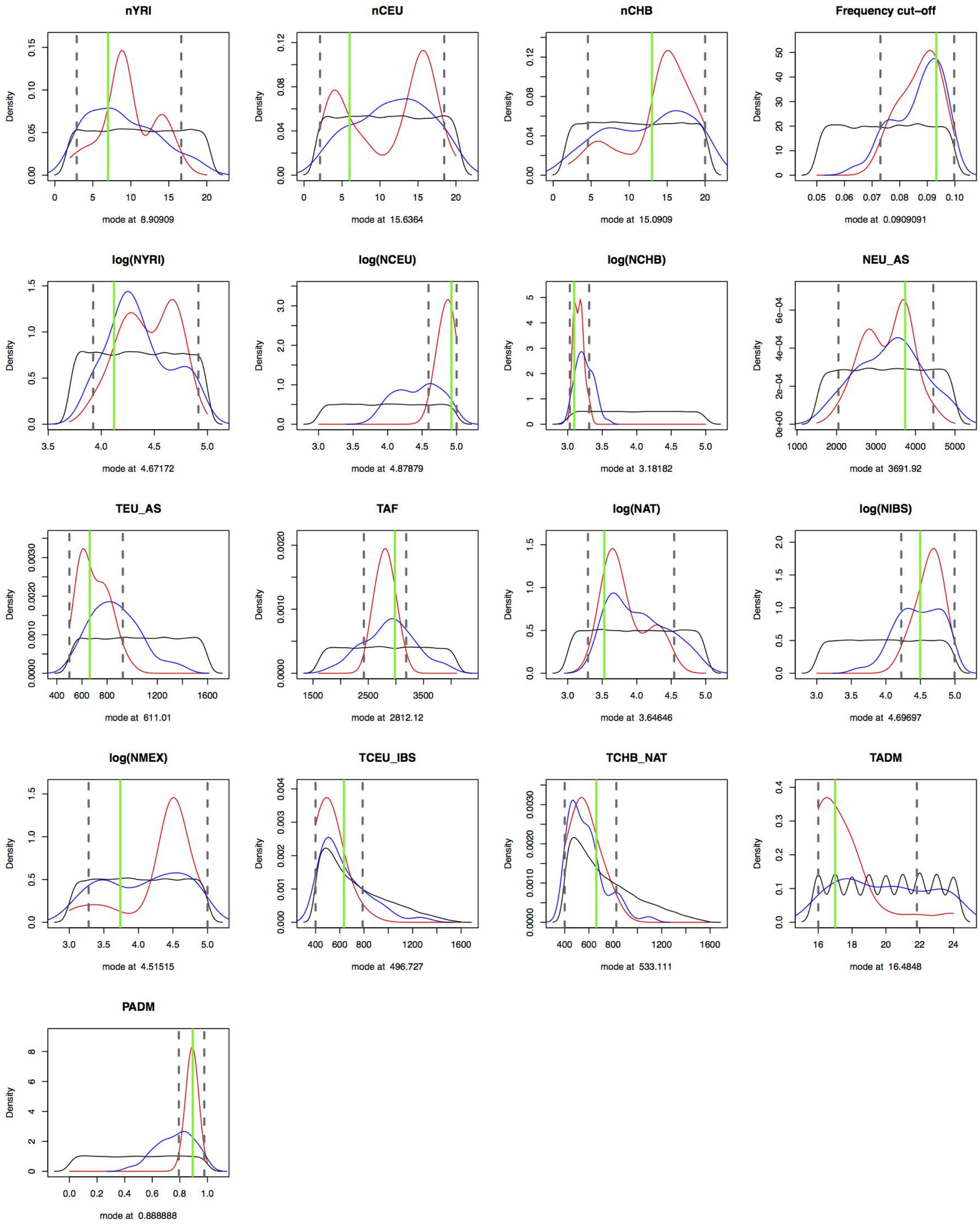

Supplementary Figure S8. Posterior distributions of the discovery set and demographic parameters of the Mexican admixture model using simulated data. Same color legend as Figure S7.

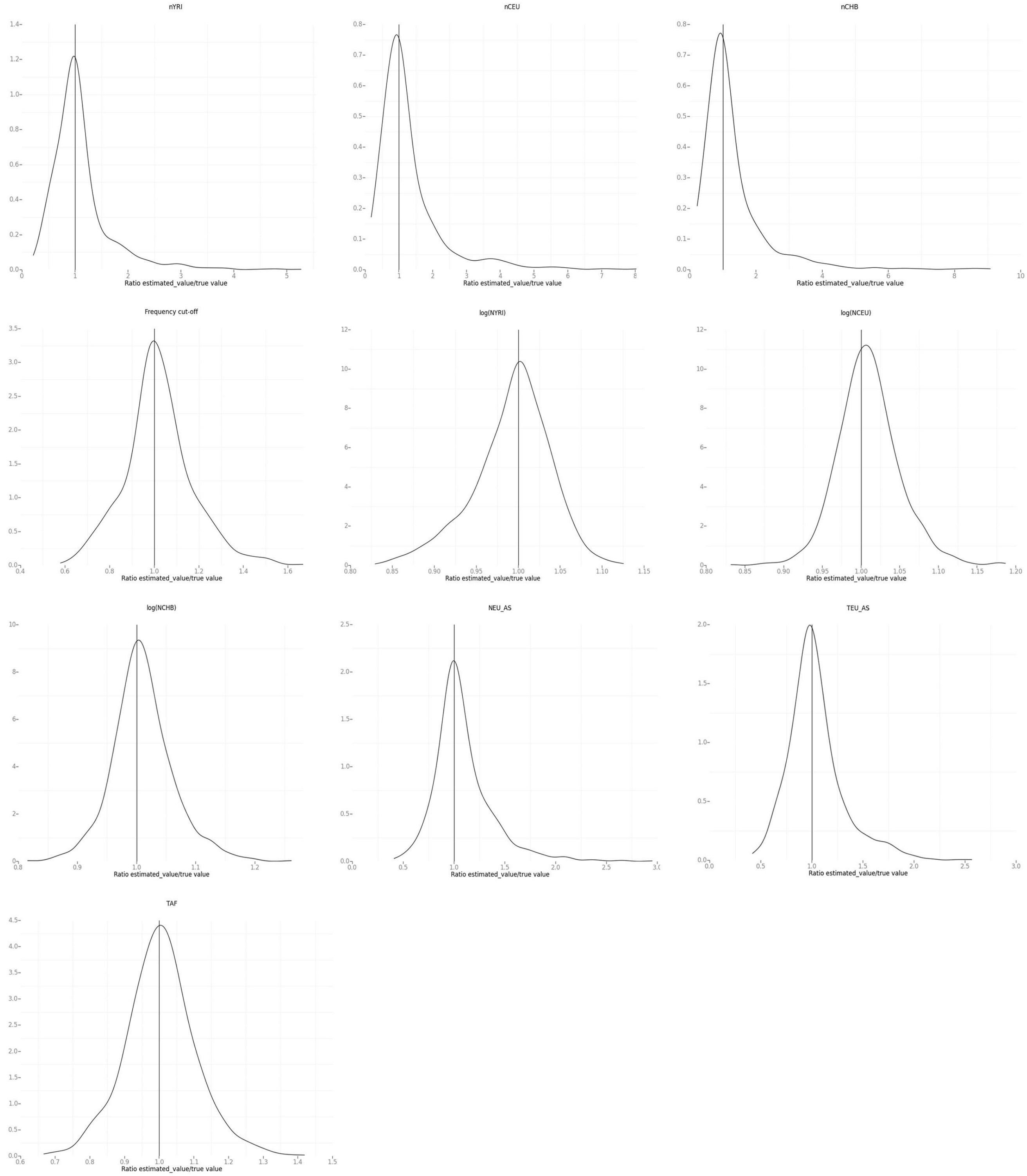

Supplementary Figure S9. **Distribution of the ratio of the estimated and the true value of the parameters of 1,000 pseudo observed datasets from the Out-of-Africa model.**

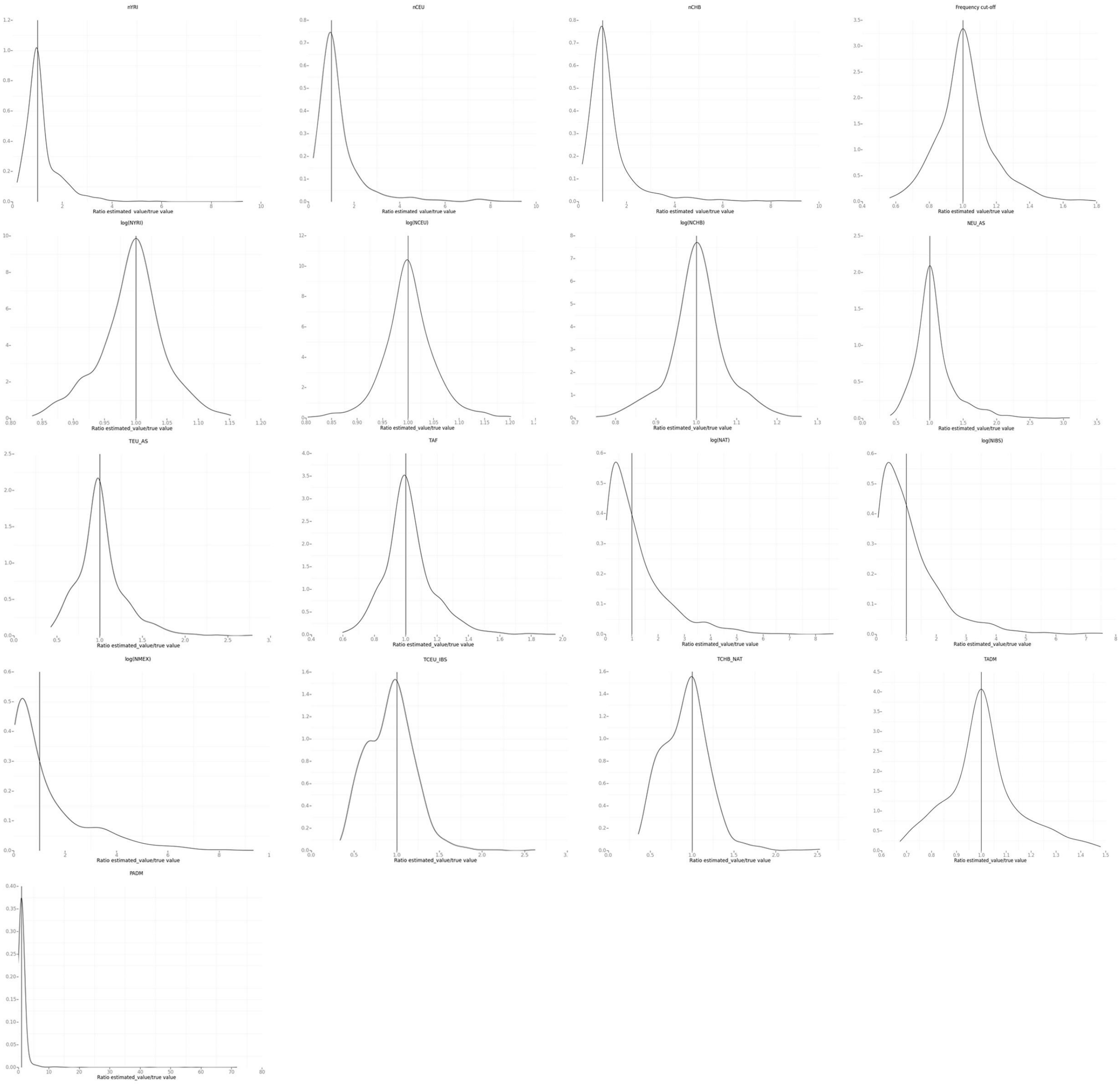

Supplementary Figure S10. **Distribution of the ratio of the estimated and the true value of the parameters of 1,000 pseudo observed datasets from the Mexican admixture model.**

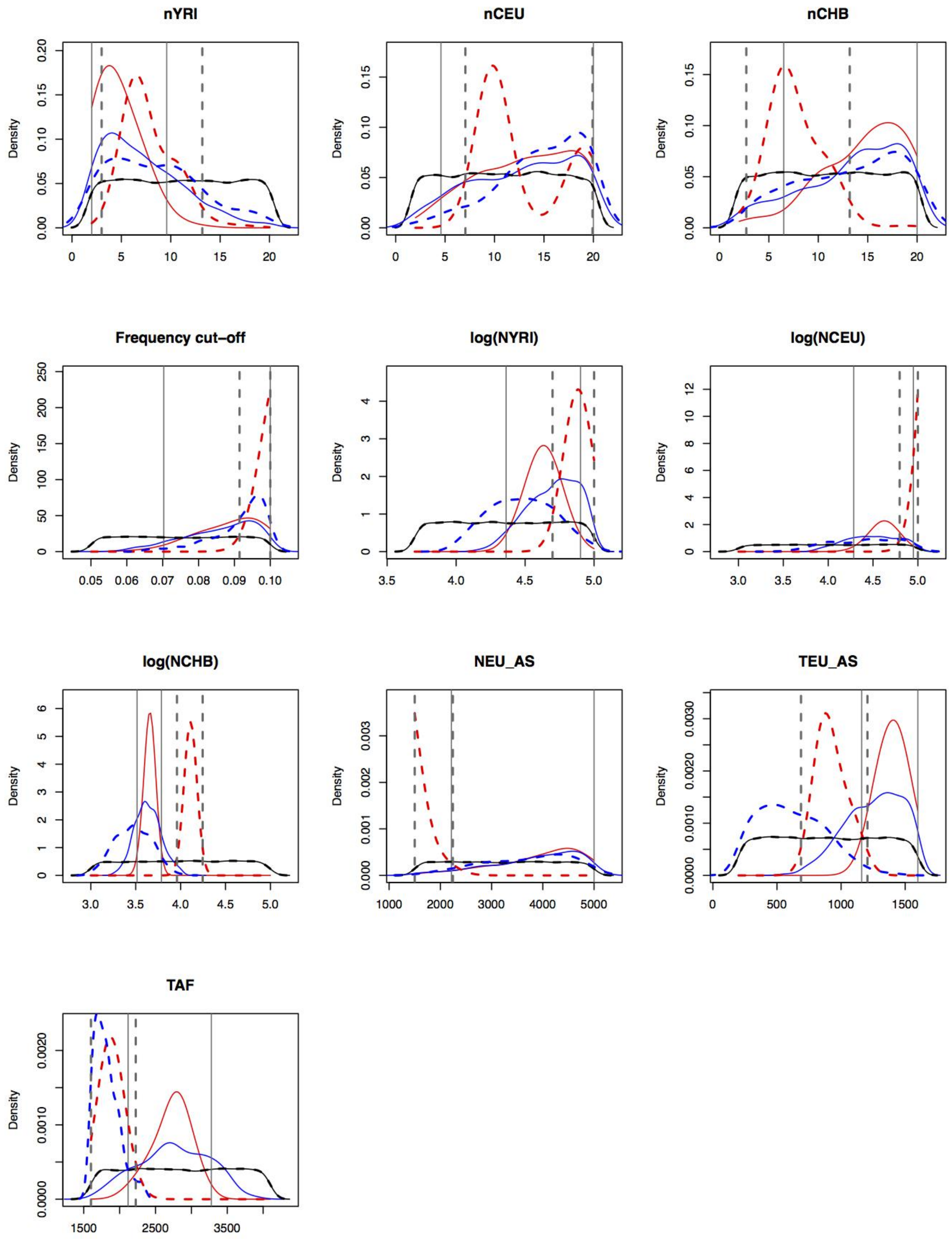

Supplementary Figure S11. Comparison of the inferred posterior distributions of the parameters of the Out-of-Africa model using observed data. The plots depict the posterior distributions obtained with Wollstein's method (dash lines) and our pipeline (solid lines) when the observed data was used. The posterior distributions are in red, the truncated priors (estimated from the retained simulations) are shown in blue, the prior distributions are drawn in black, and the vertical grey lines correspond to the 95% HPDI.

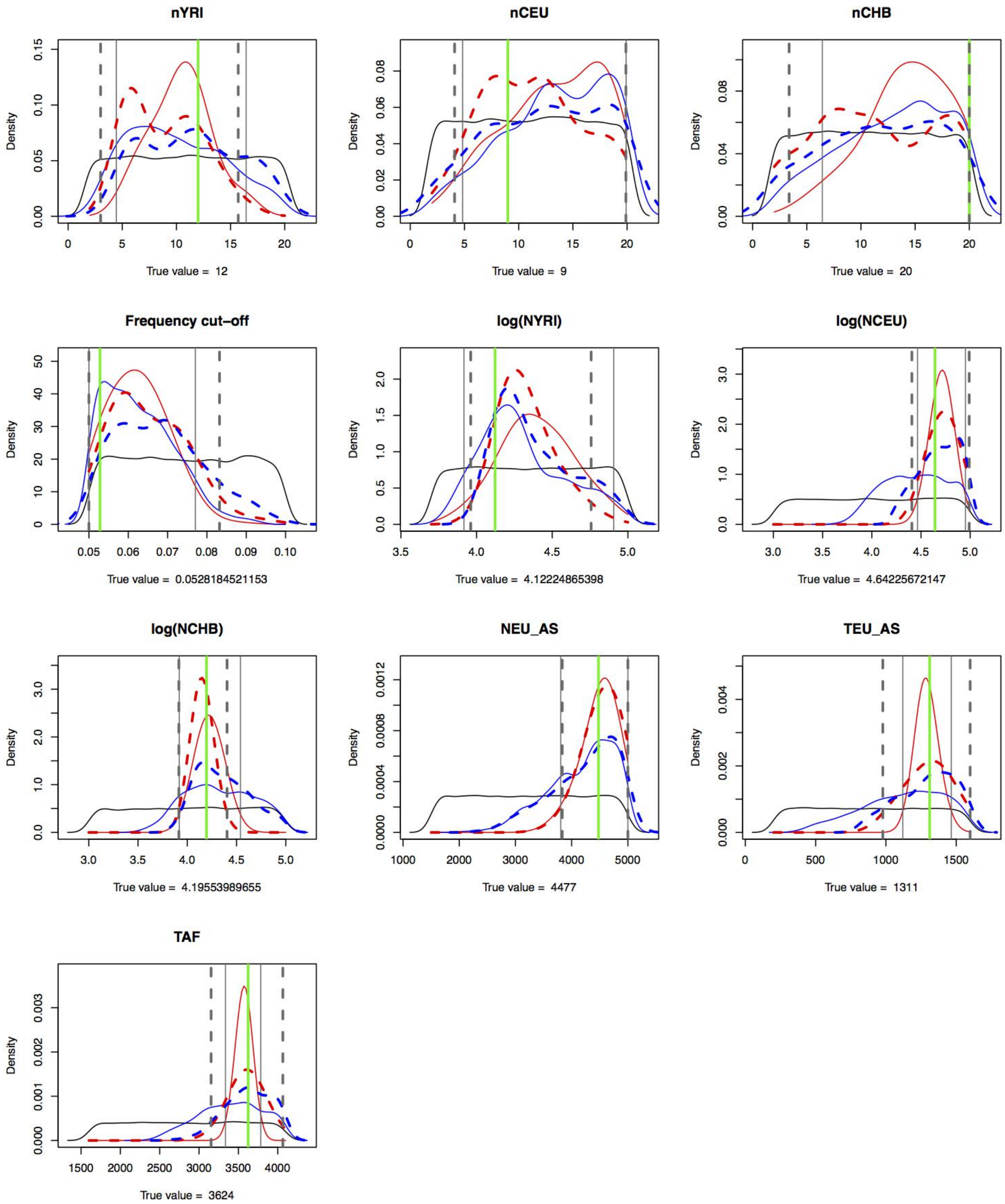

Supplementary Figure S12. Comparison of the inferred posterior distributions of the parameters of the Out-of-Africa model using simulated data.

a.

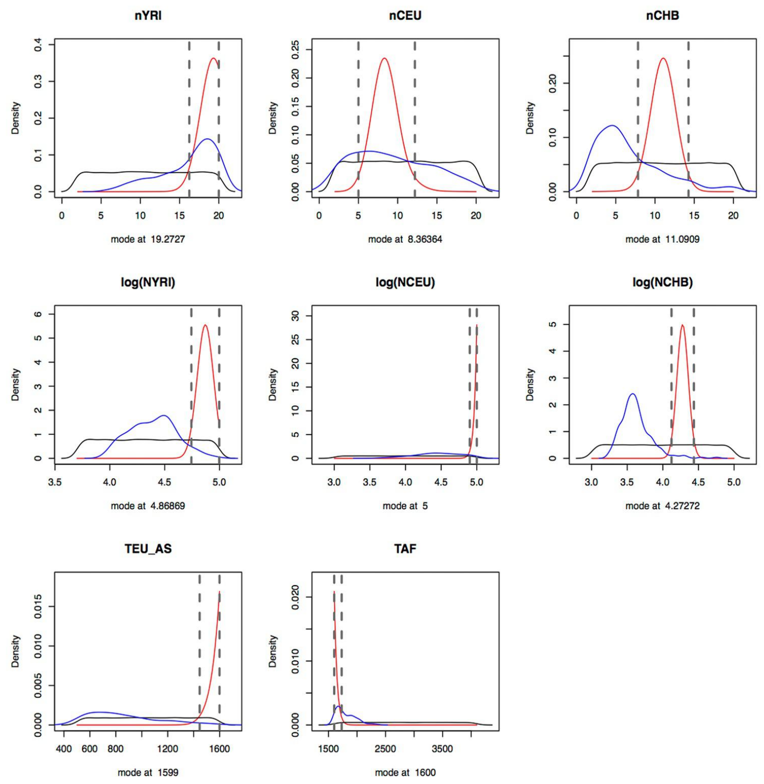

b.

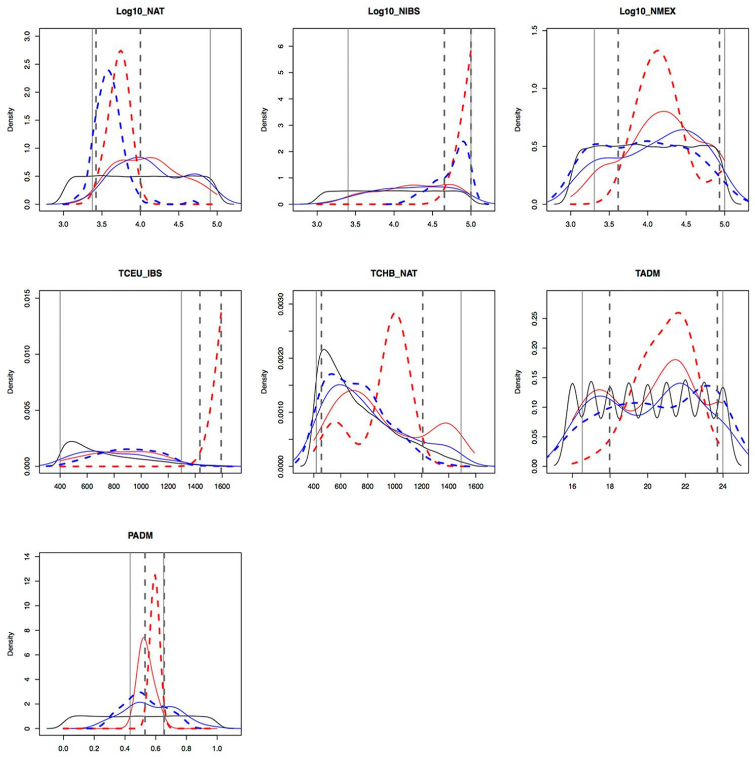

Supplementary Figure S13. Comparison of the inferred posterior distributions of the parameters of the Mexican admixture model using observed data.

a. Posterior distributions of the HapMap demographic parameters and the ascertainment parameters inferred with Wollstein's method, and used as priors for the Mexican admixture model. **b.** Comparison of the posterior distributions of the Mexican admixture model. Dash lines correspond to Wollstein's method and solid lines to our pipeline.

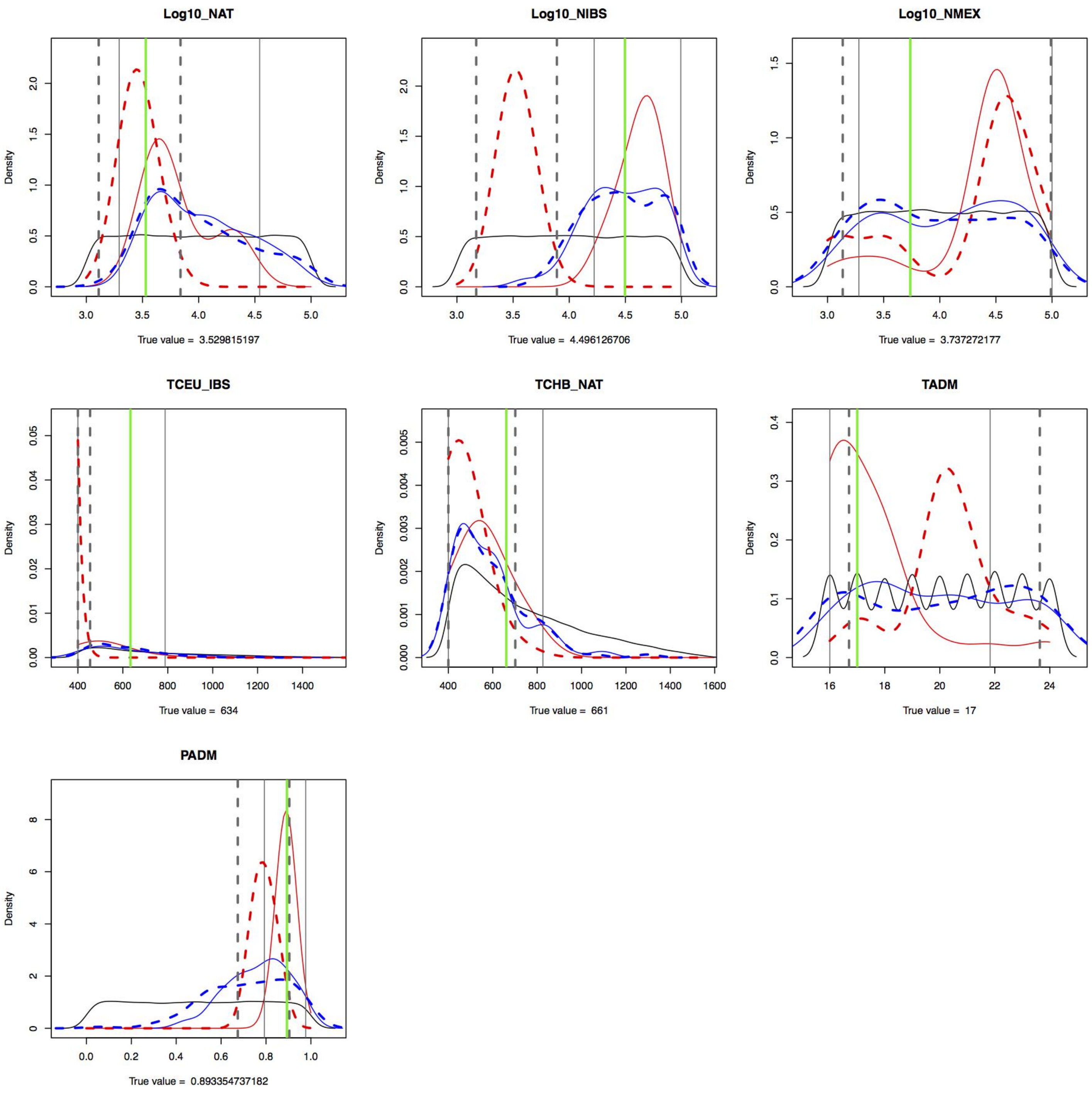

Supplementary Figure S14. Comparison of the inferred posterior distributions of the parameters of the Mexican admixture model using simulated data. Same color legend as Figure S11. The green vertical lines represent this time the true value of each of the parameters. Dash lines correspond to Wollstein's method and solid lines to our pipeline.

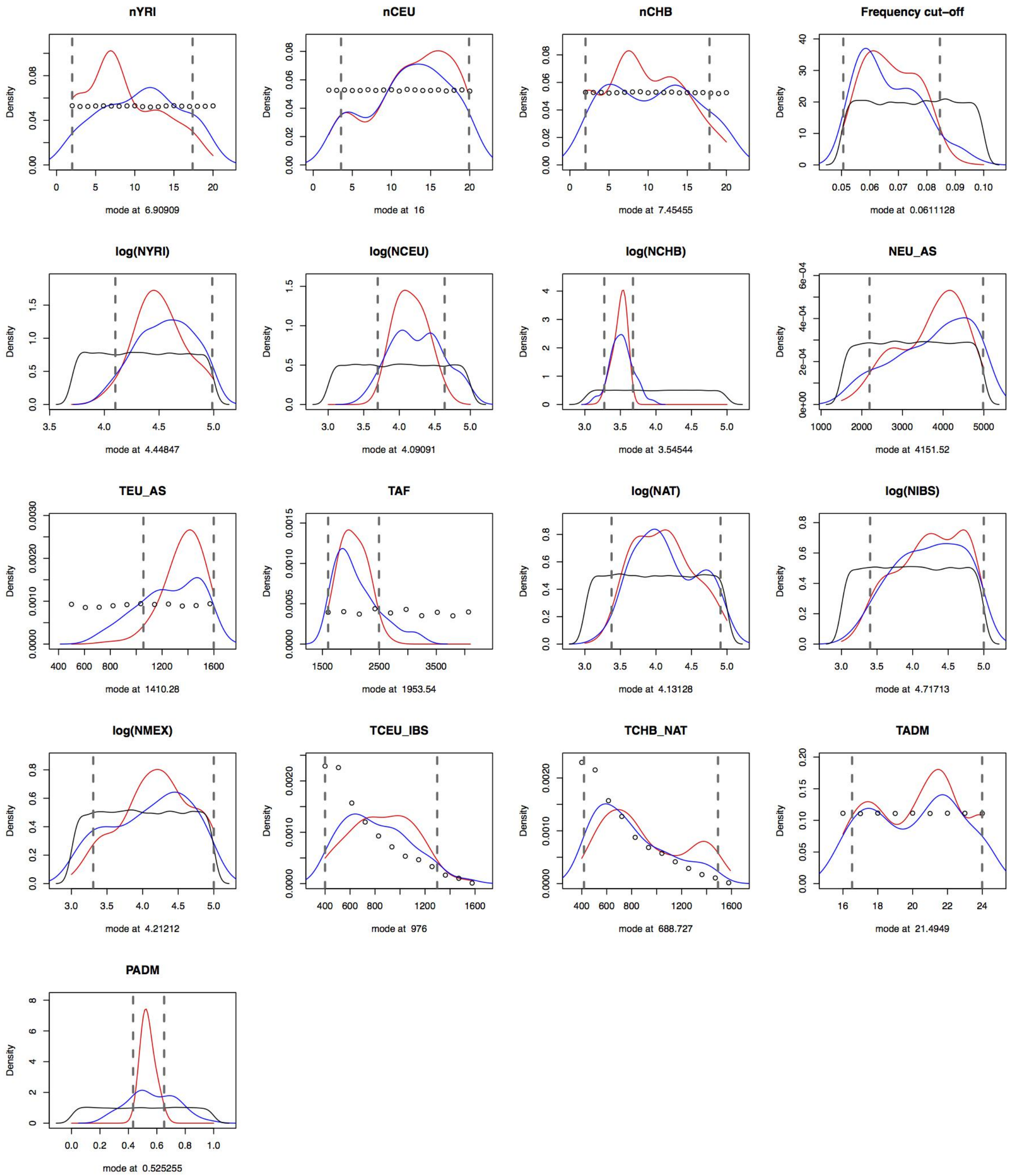

Supplementary Figure S15. Posterior distributions of the discovery set and demographic parameters as estimated from the Mexican admixture model using observed data. The black curve corresponds to the prior distribution of the parameter values, while the blue one is the truncated prior distribution. The truncated prior distribution is the distribution of the parameters kept after the rejection process (part of ABCtoolbox's method). The red curve is the posterior distribution, and the dashed lines are the 95% High Posterior Density Interval. These results were calculated using 8 PLS components and 100 retained simulations (out from 500,000 simulations).

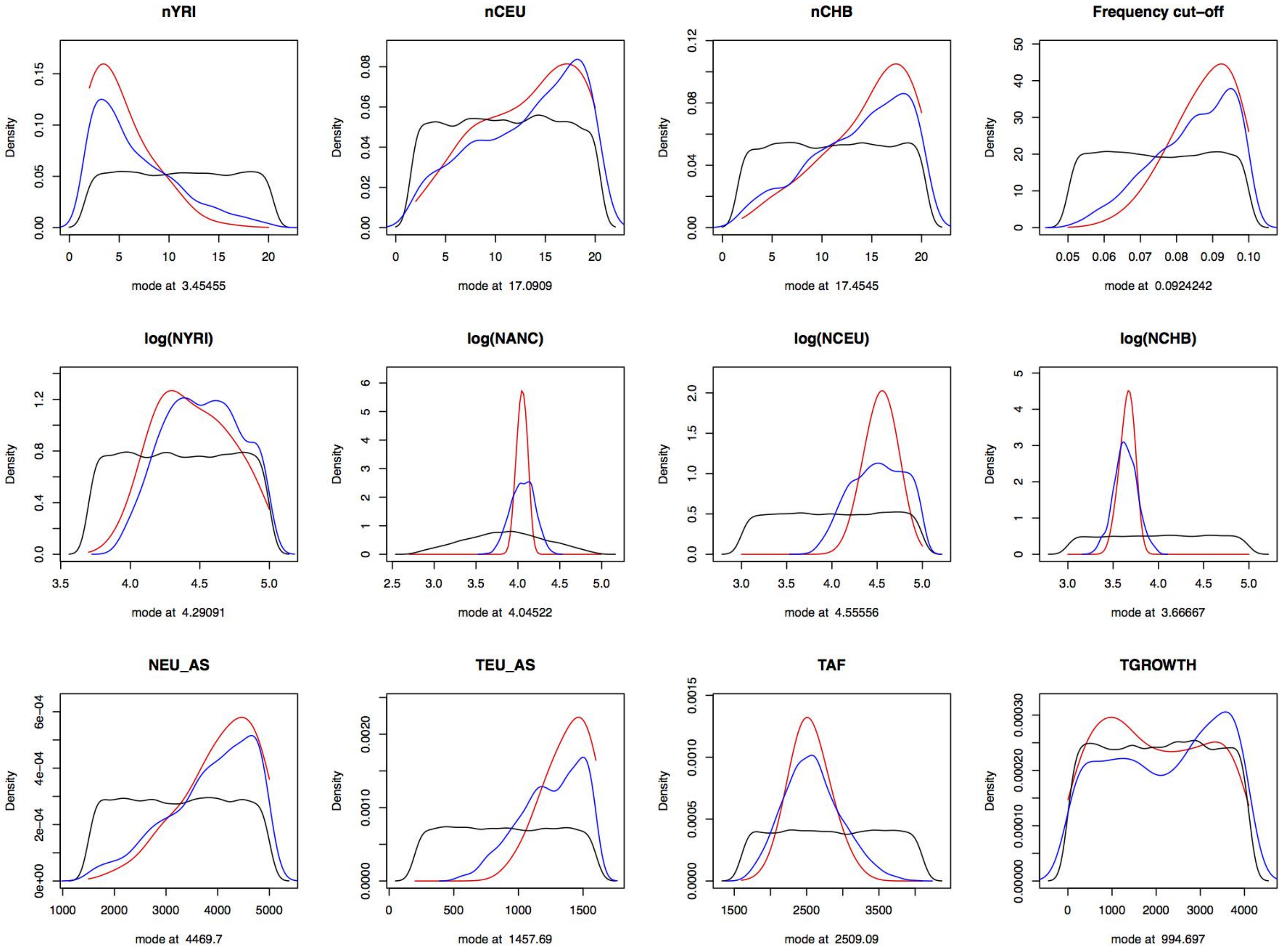

Supplementary Figure S16. Posterior distributions of Out-of-Africa model when the time of population growth in Africans is estimated. The posterior and truncated prior distributions of the time of growth in Africa have the same overall shape as the prior distribution, meaning that the simulations of the out-of-Africa model and our pipeline do not provide enough information to properly infer this particular parameter. Same color legend as Figure S15.

References

1. Arbiza, L., Zhong, E. & Keinan, A. Nre: a tool for exploring neutral loci in the human genome. *BMC Bioinforma.* **13**, 1–6 (2012).
2. Chen, G. K., Marjoram, P. & Wall, J. D. Fast and flexible simulation of DNA sequence data. *Genome Res.* **19**, 136–142 (2009).
3. Nachman, M. W. & Crowell, S. L. Estimate of the mutation rate per nucleotide in humans. *Genet.* **156**, 297–304 (2000).
4. International HapMap Consortium. A second generation human haplotype map of over 3.1 million SNPs. *Nat.* **449**, 851–861 (2007).
5. Wegmann, D., Leuenberger, C., Neuenschwander, S. & Excoffier, L. ABCtoolbox: a versatile toolkit for approximate Bayesian computations. *BMC Bioinforma.* **11**, 1–7 (2010).
6. Boulesteix, A.-L. & Strimmer, K. Partial least squares: a versatile tool for the analysis of high-dimensional genomic data. *Briefings Bioinforma.* **8**, 32–44 (2007).
7. Wegmann, D., C, L. & L, E. Efficient Approximate Bayesian Computation coupled with Markov Chain Monte Carlo without likelihood. *Genet.* **18**, 1207–1218 (2009).
8. Leuenberger, C. & Wegmann, D. Bayesian computation and model selection without likelihoods. *Genet.* **184**, 243–252 (2010).
9. Chang, C. C. *et al.* Second-generation PLINK: rising to the challenge of larger and richer datasets. *GigaScience* **4**, 1–16 (2015).
10. Price, A. L. *et al.* Principal components analysis corrects for stratification in genome-wide association studies. *Nat. Genet.* **38**, 904–909 (2006).
11. Alexander, D. H., Novembre, J. & Lange, K. Fast model-based estimation of ancestry in unrelated individuals. *Genome Res.* **19**, 1655–1664 (2009).
12. Wollstein, A. *et al.* Demographic history of Oceania inferred from genome-wide data. *Curr. Biol.* **20**, 1983–1992 (2010).
13. Drmanac, R. *et al.* Human genome sequencing using unchained base reads on self-assembling DNA nanoarrays. *Sci.* **327**, 78–81 (2009).
14. The 1000 Genomes Project Consortium. An integrated map of genetic variation from 1,092 human genomes. *Nat.* **491**, 56–65 (2012).
15. Moreno-Estrada, A. *et al.* The genetics of Mexico recapitulates Native American substructure and affects biomedical traits. *Sci.* **344**, 1280–1285 (2014).
